# Therapeutic Stress-Induced Remodeling of Transposable Elements and TE-Gene Chimeras in KYSE150 Esophageal Squamous Cell Carcinoma Cells

**DOI:** 10.3390/ijms27083471

**Published:** 2026-04-13

**Authors:** Muhammad Majid, Muhammad Moeen, Nouman Amjad, Hashim Khan, Zhaojian Sun, Linping Wu, Zhiyuan Li

**Affiliations:** 1 CAS Key Laboratory of Regenerative Biology, Guangdong Provincial Key Laboratory of Stem Cells and Regenerative Medicine, Guangzhou Institutes of Biomedicine and Health, Chinese Academy of Sciences (CAS), Guangzhou 510530, China; muhammad@gibh.ac.cn (M.M.); nouman@gibh.ac.cn (N.A.); khanhashim@gibh.ac.cn (H.K.); sun_zhaojian@gibh.ac.cn (Z.S.); wu_linping@gibh.ac.cn (L.W.); 2 Department of Bioinformatics, Faculty of Chemical and Biological Sciences, The Islamia University of Bahawalpur, Bahawalpur 63100, Pakistan; muhammadmoeen416@gmail.com; 3 Department of Pathology, Faculty of Veterinary and Animal Sciences (FV&AS), The Islamia University of Bahawalpur, Bahawalpur 63100, Pakistan

**Keywords:** TEs, chimeric transcripts, ESCC, 125I radiation, carfilzomib

## Abstract

Transposable elements (TEs) are major contributors to genome plasticity and can reshape gene regulation through stress-responsive activation and the formation of TE-gene chimeric transcripts. Although therapeutic stress is known to perturb transcriptional networks in cancer cells, its impact on canonical TE transcription and TE-gene chimera formation in esophageal squamous cell carcinoma (ESCC) remains poorly defined. To address this, we performed a comprehensive transcriptome-wide analysis of TE expression and TE-gene chimeric transcripts in KYSE150 ESCC cells following combined 125I radiation and carfilzomib treatment. The TE analysis showed 148 dysregulated TEs, characterized by ERV1 LTR element enrichment and distinct treatment-control sample separation, indicating structured remodeling of the TE transcriptome. We identified 301 significant TE-gene chimeric events, indicating category-specific remodeling with an increase in TE-initiated and TE-exonic chimeras and a decrease in TE-terminal events. The TE families that underwent the most transcriptional changes were not those that drove chimeric events, indicating that global TE activation does not passively cause chimera remodeling. The gene repression was strongly associated with chimeric transcripts, and gene expression changes were negatively correlated with chimerism frequency. *SPANXN1*, *IL1RL1*, and *RSAD2*, strongly downregulated genes, produced novel TE-derived isoforms and were high-potential functional candidates. Epigenetic context analysis showed considerable overlap between exonized chimeras and candidate cis-regulatory elements, suggesting a potential association with regulatory genomic contexts. Pathway enrichment analysis showed synchronized transcriptomic reprogramming and cell cycle and DNA repair pathway activation and autophagy inhibition. In esophageal cancer cells, concurrent genotoxic and proteotoxic stress causes complex TE remodeling, linking traditional TE transcriptional alterations to structured TE-gene chimera development and stress-related transcriptome reprogramming.

## 1. Introduction

ESCC is a highly aggressive malignancy, representing the predominant subtype of esophageal cancer, and is associated with unfavorable clinical outcomes due to delayed diagnosis and restricted treatment options [1,2,3]. The KYSE-150 cell line was established from a poorly differentiated ESCC of the upper esophagus of a patient who had already received treatment. It serves as a well-established in vitro model for studying ESCC biology and how it responds to treatment [4,5,6,7]. KYSE-150 also has a high tumor mutation burden (about 70.4 mutations per megabase) and a strong ability to invade, which makes it a very important cell line for studying ESCC [2,8,9].

TEs, long considered merely a source of so-called junk DNA, have become known as powerful regulators of genome activity [10,11,12]. TEs constitute approximately half of the human genome [13,14], and these mobile elements can transpose themselves, modify genomic structure, and change transcriptional programs, all of which are common features of cancer [15,16,17]. In normal cells, TE activity is regulated through silencing mechanisms, which involve the activation of DNA methylation and histone modification [18,19,20]. In the event of cellular stress (e.g., radiation or drug treatment), TEs may be activated and result in differential expression and the production of TE-gene chimeric transcripts, which can confer novel regulatory potential and/or phenotypic consequences on tumor cells [21,22,23]. Such aberrant transcripts may rewire oncogenic pathways by onco-exaptation, act as tumor-specific neoantigens presented by the MHC-I, and induce innate immune responses via cytosolic nucleic acid sensing [23,24]. Although increasing attention is being given to TE activation in the context of stress, there is a lack of characterization of the dynamics of TE activation in ESCC, particularly under combined treatment paradigms.

Localized radiation for ESCC is one of the treatment options that can be performed with 125Iodine seed brachytherapy [25,26]. Even though it causes reactive oxygen species (ROS), DNA damage, and apoptosis through endoplasmic reticulum stress (ERS) and the unfolded protein response (UPR), its effectiveness is limited by the fact that it is naturally resistant to radioactivity [1,27,28,29]. Carfilzomib (CFZ) is an irreversible proteasome inhibitor that has been reported to disrupt protein homeostasis, induce endoplasmic reticulum stress (ERS), and inhibit endoplasmic reticulum-associated degradation (ERAD) pathways [30,31]. Recent research indicates that the combination of CFZ and 125I seed radiation significantly enhances ESCC cell death, particularly through apoptosis, paraptosis, and ferroptosis, by inducing heightened cellular stress (e.g., ERS, UPR), increasing the production of reactive oxygen species (ROS), causing calcium (Ca^2+^) overload, promoting protein ubiquitylation, and inhibiting ferroptosis (e.g., GPX4, SLC7A11) [1,32]. Nonetheless, the impact of this concurrent genotoxic and proteotoxic stress on the restructuring of TE transcriptional landscapes and the facilitation of TE-gene chimeric transcript formation remains to be systematically investigated.

Prior research has predominantly concentrated on mechanisms of cell death; however, the transcriptional effects of concurrent 125I and carfilzomib treatment on transposable elements in ESCC are not yet elucidated. We posit that the interplay of genotoxic and proteotoxic stress catalyzes structured reconfiguration of TE transcription and promotes TE-gene chimera formation, thereby facilitating stress-induced transcriptome reprogramming in ESCC. This study, therefore, systematically profiles both canonical TE expression and TE-gene chimeric transcripts in KYSE150 cells following combined 125I and carfilzomib treatment. We conducted an extensive transcriptome-wide analysis of high-throughput RNA-sequencing data from KYSE150 cells to measure repetitive element expression and identify TE-gene chimeric fusions. This study seeks to clarify the impact of therapeutic stress on TE activity and transcript architecture in ESCC by combining differential expression, genomic context, and functional enrichment analyses. Ultimately, our research aims to delineate TE-mediated transcriptomic remodeling as a potential element of the therapeutic stress response and a source of novel regulatory candidates in esophageal cancer.

## 2. Results

### 2.1. Stress Induces Remodeling of TE Transcription

**Figure 1:**
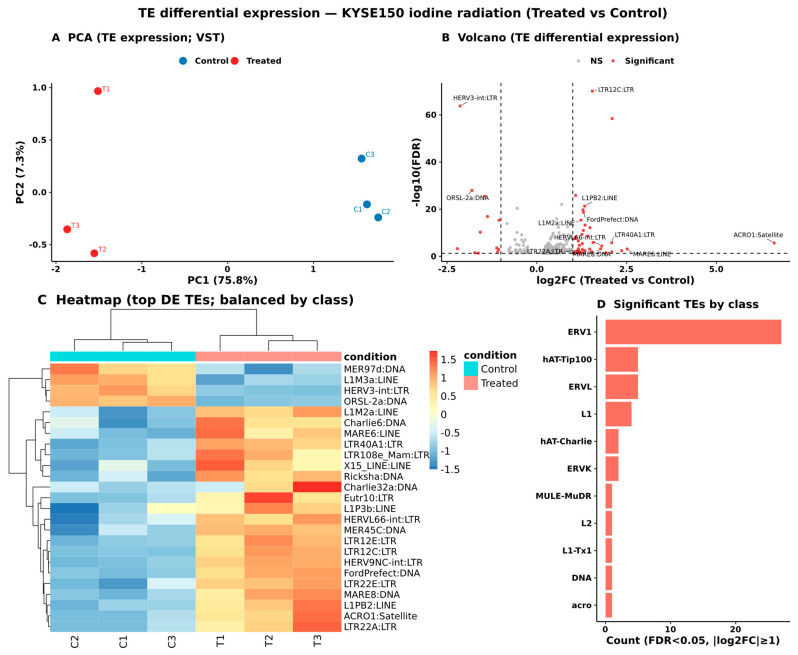
Iodine radiation induces selective remodeling of the transposable element landscape in KYSE150 cells. (**A**) Principal component analysis (PCA) of variance-stabilized TE/repeat expression values derived from TEtranscripts count data. (**B**) TEtranscripts differential expression results (treated vs. control) are displayed as a volcano plot with log2FC versus −log10 padj (FDR). Representative elements are labeled to highlight significantly deregulated TE/repeat features (FDR < 0.05, |log2FC| ≥ 1). (**C**) Hierarchical clustering heatmap of top differentially expressed TE/repeat features, balanced by TE class representation, illustrating coordinated upregulation and downregulation patterns across biological replicates. (**D**) Distribution of significantly deregulated TE/repeat features stratified by TE class.

TE differential expression analysis showed that iodine radiation caused a considerable change in the TEs landscape in KYSE150 esophageal cancer cells. Using a strict criterion (FDR < 0.05 and |log2FC| > 1), we found 148 TEs that were significantly deregulated. This shows that there is a systematic and reproducible repeat transcriptional response. Principal component analysis of variance-stabilized TE expression patterns exhibited distinct separation between treated and control samples, thereby validating that radiation exposure generates a coherent alteration in the TE transcriptome (Figure 1A). The volcano plot clearly showed that many TE features had large effect sizes and were statistically significant (Figure 1B). Hierarchical clustering of the most differentially expressed TE/repeats, evenly distributed among TE classes, showed coordinated patterns of upregulation and downregulation across biological replicates. This further supports the idea that repeats respond to radiation in a selected way rather than randomly (Figure 1C). Class-level quantification showed that retrotransposon families were significantly enriched among differentially expressed elements. The most affected subclass was ERV1 LTR elements (*n* = 27). The ERVL (*n* = 5) and ERVK (*n* = 2) elements followed, along with deregulated LINE elements such as L1 (*n* = 4) (Figure 1D). DNA transposon subclasses, such as hAT-Tip100 (*n* = 5) and hAT-Charlie (*n* = 2), were also significant. These findings suggest that iodine radiation has a significant impact on retrotransposon-derived elements, especially ERV1 LTR sequences. This suggests that stress changes the transcriptional activity of endogenous retroviruses in KYSE150 cells.

### 2.2. Iodine Treatment Induces Category-Specific TE-Gene Chimera Formation

**Figure 2:**
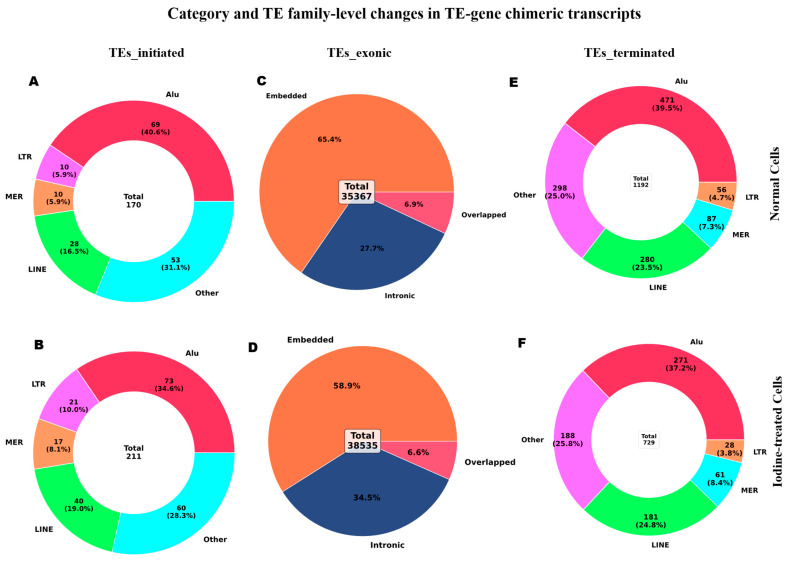
Combined treatment induces category-specific remodeling of TE–gene chimeric transcripts in KYSE150 cells. (**A**,**B**) Distribution of TE-initiated chimeric transcripts in control and treated cells, including family-level composition. (**C**,**D**) Distribution of TE-exonic chimeric transcripts in control and treated cells, including subclassification into embedded, overlapped, and intronic events. (**E**,**F**) Distribution of TE-terminal chimeric transcripts in control and treated cells, including family-level composition. Total event counts are shown for each category to illustrate treatment-associated shifts in chimera abundance and composition.

Our comprehensive analysis revealed that combined 125I radiation and carfilzomib treatment induces significant alterations in TE-gene chimeric transcript formation in KYSE150 esophageal cancer cells. In contrast to TE-terminal types, which decreased from 1192 to 729 transcripts, TE-initiated types increased from 170 to 211 and TE-exonic types expanded from 35,367 to 38,535 transcripts (Figure 2A–F). Family-level characterization revealed nuanced activation patterns; TE-initiated types were more involved in LINE (28 to 40), LTR (10 to 21), and MER (10 to 17) elements (Figure 2A,B). Differential dynamics were revealed by a detailed analysis of exonized types: embedded types dropped from 23,119 to 22,710, overlapped types increased from 2456 to 2540, and intronic types increased from 9792 to 13,285 (Figure 2C,D). A more detailed breakdown of TE family distributions within each exonized subtype (embedded, overlapped, and intronic) is provided, highlighting subtype-specific shifts between control and treated conditions (Figures S1–S3).

In all categories, we found 301 statistically significant TE-chimeric events (FDR < 0.05), with the most commonly involved families being AluSx (24 genes), AluJb (20 genes), MIRb (18 genes), and SINE elements emerging as dominant drivers (Figure S4). In addition to identifying significant events, differential expression analysis of TE-gene chimeras revealed a predominance of strongly downregulated transcripts following treatment. These events were inferred from short-read RNA-seq data and therefore do not fully resolve complete transcript structures or precise breakpoint positions. Enhanced volcano plot visualization highlighted top suppressed candidates including *SPANXN1* (log2FC = −6.70), *IL1RL1* (log2FC = −6.15), and *RSAD2* (log2FC = −5.63) (Figure S5), indicating that TE incorporation frequently coincides with marked repression of specific associated genes.

### 2.3. Treatment Expands TE-Gene Chimeric Repertoires and Reveals Distinct Association Patterns

**Figure 3:**
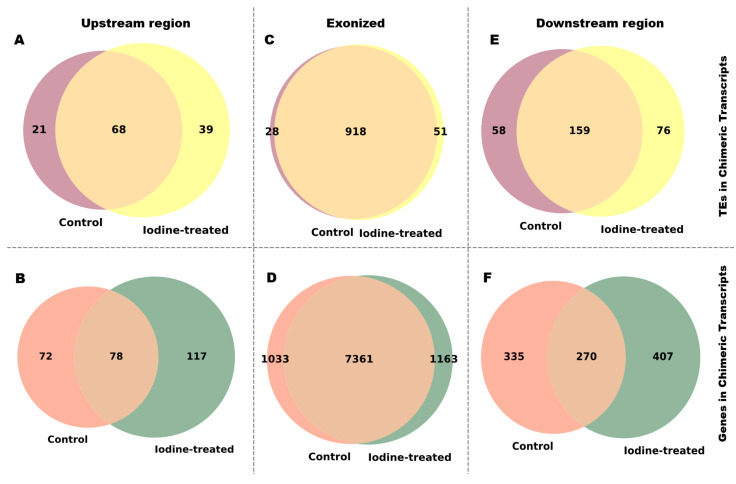
Venn diagrams of TEs and genes involved in chimeric transcript formation. The Venn diagrams depict the unique number of TEs and genes involved in chimeric transcript formation in normal and treated conditions across all three types: TE-initiated, Exonic, and TE-terminal. TE-initiated chimeras are represented by (**A**,**B**), where TEs are shown in (**A**) and genes in (**B**). Exonized chimeras are shown in (**C**,**D**), with TEs in (**C**) and genes in (**D**). Finally, TE-terminal chimeras are represented by (**E**,**F**), with TEs in (**E**) and genes in (**F**). The numbers indicate the unique and shared counts found in the control and iodine-treated samples.

**Figure 4:**
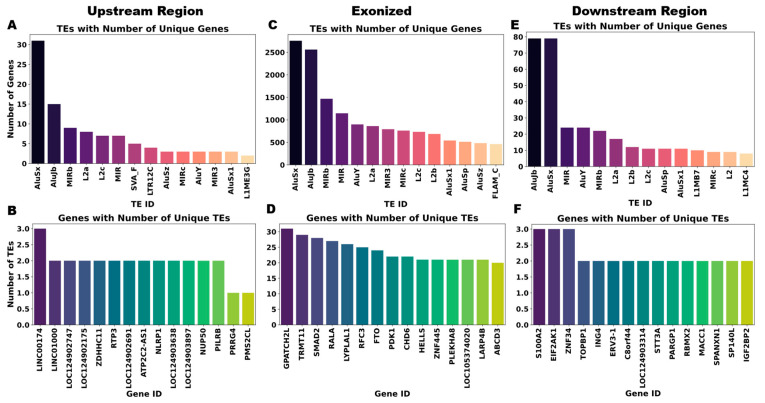
Distinct partner selection patterns of TEs and genes in chimeric transcript formation. The TE-initiated chimeras (**A**) demonstrated that AluSx, AluJb, and MIRb had a broad interaction capacity with multiple gene partners, whereas the gene-initiated chimeras (**B**) exhibited restricted TE associations, such as *LINC00174* and *LINC01000* forming limited partnerships. TE-exonic chimeras (**C**) exhibited extensive partner diversity, with Alu families contributing to thousands of unique gene fusions. In contrast, select genes, including *GPATCH2L*, *TRMT11*, and *SMAD2* (**D**), interacted broadly across many TE families. In TE-terminal chimeras (**E**), AluSx and AluJb partnered with numerous genes, in contrast to the more limited associations of MIR. Gene partners in this category (**F**), such as *S100A2*, *EIF2AK1*, and *ZNF34*, exhibited relatively uniform TE engagement.

Venn diagram analysis revealed treatment-induced expansion of TE and gene involvement across all chimera categories. TE-initiated chimeras exhibited enhanced compositional diversity, with unique TEs increasing from 21 to 39 and unique genes rising from 72 to 117 (Figure 3A,B). Exonized chimeras showed more participation, with unique TEs going up from 28 to 51 and gene partners going up from 1033 to 1163 (Figure 3C,D). TE-terminal chimeras showed elevated involvement of both TEs (58 to 76) and genes (335 to 407) despite overall count reductions (Figure 3E,F). Analysis of chimeric pairing patterns revealed marked asymmetry in association breadth. AluSx, AluJb, and MIRb families exhibited high connectivity, forming chimeric transcripts with numerous gene loci (Figure 4A,C,E). On the other hand, genes usually had limited chimeric repertoires, each linking to only a small number of TE families (Figure 4B,D,F). This prevalent hub-like behavior, especially among SINE elements, indicates a mechanism for instant transcriptome diversification in response to genotoxic stress conditions.

### 2.4. Formation of TE-Gene Chimeric Transcripts Is Associated with Expression Changes in Associated Genes

**Figure 5:**
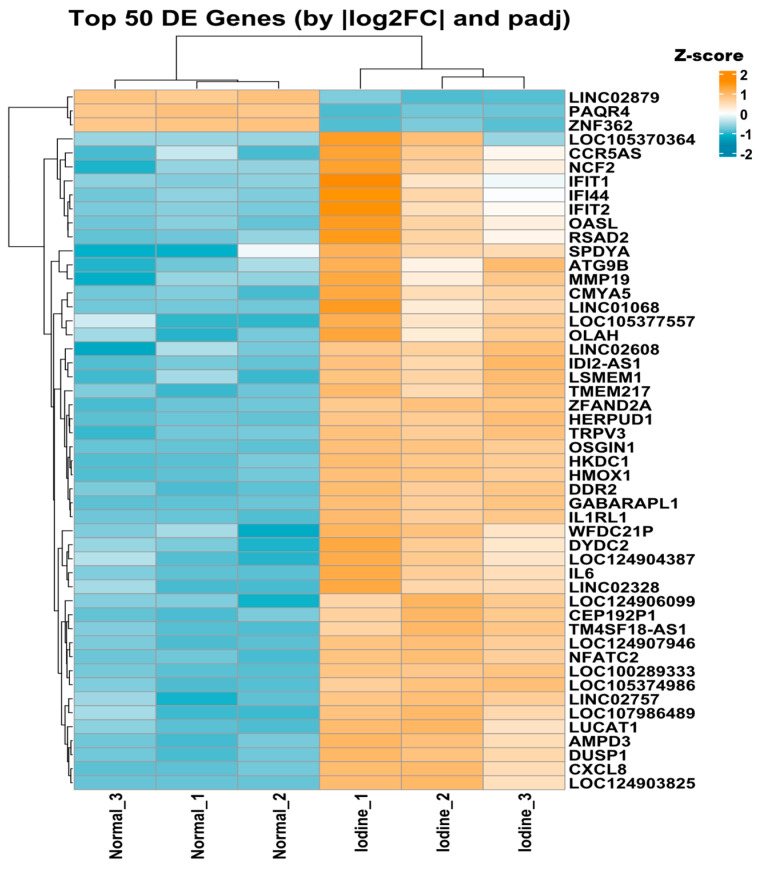
DEGs associated with chimeric transcript formation after treatment. The heatmap displays the top 50 DEGs associated with TE_exonic type of chimeric transcripts with respect to log2FC and padj values.

We conducted differential expression analysis for genes associated with TE-initiated, TE-terminal, and TE-exonic chimeric transcripts following treatment with iodine and carfilzomib. The results revealed 1460 differentially expressed genes (DEGs) in the TE-exonic category, including 779 upregulated and 681 downregulated genes; among them, the top 50 DEGs are shown in the heatmap (Figure 5). In the TE-terminal (downstream) category, 112 DEGs were identified, of which 52 were upregulated and 60 were downregulated (Figure S6). For TE-initiated transcripts, we observed 42 DEGs, evenly split between 21 upregulated and 21 downregulated. To visualize these patterns, we generated a heatmap of all the DEGs for the TE-initiated type, ranked according to log2FC and padj (Figure S7).

**Figure 6:**
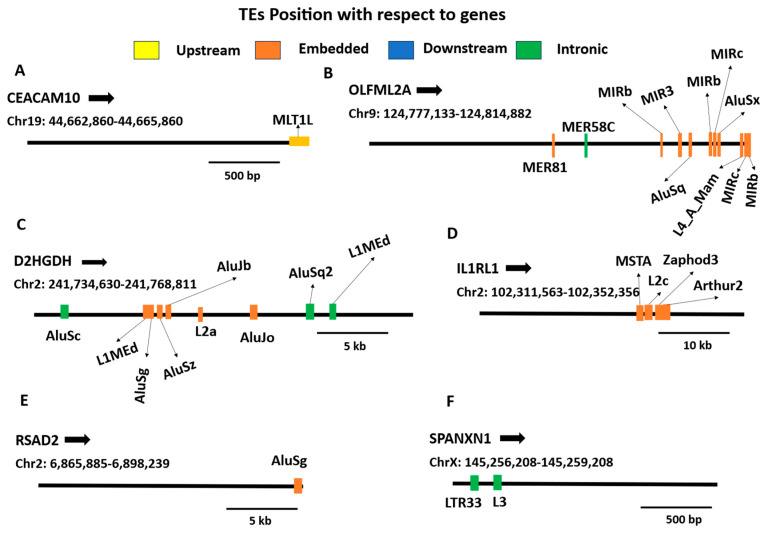
Genomic distribution of TEs associated with gene loci. Genomic positions of TE-derived sequences relative to selected gene regions are shown for (**A**) *CEACAM19*, (**B**) *OLFML2A*, (**C**) *D2HGDH*, (**D**) *IL1RL1*, (**E**) *RSAD2*, and (**F**) *SPANXN1*. Black horizontal bars represent the genomic span of each gene locus. Colored vertical ticks indicate individual TEs positioned relative to the gene body and are classified as upstream (yellow), embedded/exonic (orange), intronic (green), or downstream (blue). TE labels are displayed above or below the gene track to minimize overlap. Scale bars indicate genomic distance for each locus. Arrows denote gene orientation based on annotated strand information.

We further analyzed the 42 DEGs involved in TE-initiated chimeric transcript formation to determine whether their expression changes were associated with TE-driven transcriptional initiation, focusing on genes with the highest log2FC values. Following treatment, a chimeric transcript originating from upstream MLT1L sequences was detected at the *CEACAM19* locus, whereas no such event was observed under control conditions. This event was accompanied by significant downregulation of *CEACAM19*, supporting a potential association between TE-derived promoter activity and reduced canonical transcript expression. Similarly, after treatment, ARB2BP formed a chimeric transcript with an upstream L2a sequence that was absent in control cells, and this was also associated with significant downregulation of ARB2BP expression (Figure 6A, Table S1). The upstream TEs exhibited the highest motif densities. Alu and L1 subfamilies showed strong enrichment for KLF (KLF3, KLF5, KLF17) and ZNF family motifs, whereas PRDM9, POU factors, and FOXC2 motifs occurred at lower frequencies. Correlation analysis revealed strong co-occurrence patterns among KLF motifs and moderate clustering among ZNF motifs, while PRDM9 and NR3C2 displayed distinct occurrence patterns. These findings suggest that upstream TEs may function as regulatory hotspots, where coordinated KLF and ZNF motif enrichment could influence gene expression programs (Figure S8).

We next examined 50 DEGs associated with exonization-type TE chimeras (25 upregulated and 25 downregulated genes). The *OLFML2A* gene exhibited multiple TE-containing chimeric isoforms derived from Alu, MIR, L4, and MER elements. Isoforms containing AluSq, AluSx, MIRb, and MIRc were present in both control and treated samples but showed reduced read support following treatment. In contrast, MER58C and MER81-derived isoforms were detected only in control samples and were completely absent after treatment. Despite the selective reduction or loss of several TE-derived isoforms, overall *OLFML2A* expression was significantly upregulated (log2FC = +3), suggesting that treatment preferentially promotes expression of canonical or non-MER-containing transcripts (Figure 6B, Table S1).

*D2HGDH* also displayed extensive TE associations, particularly with Alu and LINE elements. Multiple Alu-derived chimeras (AluJb, AluJo, AluSg, AluSq2, and AluSz) were present exclusively in control cells and were lost after treatment. Conversely, new isoforms containing AluSc and an intronic L1MEd insertion emerged specifically in treated samples. Chimeras involving L1MEd and L2a were detected in both conditions but with variable read support. While the composition of TE-associated isoforms changed a lot between conditions, *D2HGDH* expression went up a lot (log2FC = +2.6). This suggests that dynamic TE isoform turnover may happen with transcriptional activation instead of repression (Figure 6C, additional file DEG_ChimericTranscript_Exonized.csv).

*IL1RL1* gene expression was strongly reduced (log2FC = −6.1, adjusted *p* < 0.001). No TE-gene chimeras were detected in control cells; however, multiple novel isoforms containing Arthur2, L2c, MSTA, and Zaphod3 elements appeared exclusively in treated samples. The emergence of these TE-derived isoforms coincided with marked transcriptional repression of *IL1RL1*, suggesting a potential disruptive effect of TE exonization on gene expression (Figure 6D). Similarly, *RSAD2*, an interferon-stimulated antiviral gene, was substantially downregulated (log2FC = −5.6, adjusted *p* < 0.001). A novel isoform carrying an embedded AluSg element emerged only after treatment and was absent in control samples, coinciding with reduced gene expression (Figure 6E). *SPANXN1* was also strongly suppressed (log2FC = −6.7, adjusted *p* < 0.001). Newly detected isoforms incorporating downstream L3 and LTR33 sequences were observed exclusively in treated cells, raising the possibility that TE-associated readthrough or altered transcript termination contributes to gene repression (Figure 6F, Table S2). We next developed a functional impact scoring framework to prioritize TE-chimeric events most likely to disrupt associated gene function. This scoring identified 16 high-impact candidates (score > 0.8). Notably, events combining strong downregulation (log2FC < −2) with SINE insertions produced the highest scores (~0.9), consistent with possible transcript-disruptive scenarios, including altered splicing or reduced transcript stability; however, these interpretations were not experimentally tested in the present study. (Figure S9, Table S3). A complete list of TE-associated isoforms is provided, including all TE-initiated DEGs, the top 50 exonized DEGs, and the top 30 TE-terminal DEGs, with their detection and expression changes across control and treated conditions (Tables S1 and S2, and DEG_ChimericTranscript_Exonized.csv).

### 2.5. Relationship Between Gene Expression Changes, Chimeric Frequency, and Chromosomal Distribution

**Figure 7:**
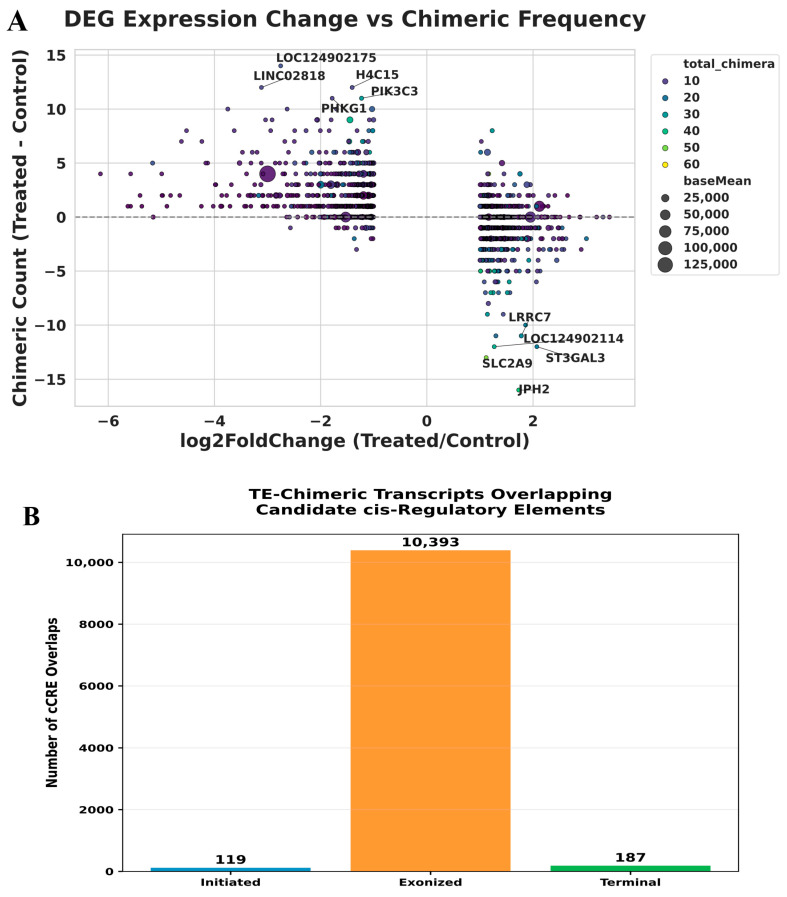
Differential expression abundance versus chimeric transcript abundance and overlap of TE-gene chimeric transcripts with candidate cis-regulatory elements (cCREs). Scatter plot showing the relationship between gene expression changes (log2FC, treated vs. control) and chimeric transcript shift counts. Each point represents a gene; point size reflects gene expression level (baseMean), and color indicates the total number of chimeric events per gene, as shown in the color scale. (**A**). Bar plot showing the number of TE-gene chimeric events overlapping ENCODE candidate cis-regulatory elements across chimera categories (initiated, exonized, and terminal). Numbers above bars indicate total overlap counts (**B**).

We next examined the relationship between differential gene expression and the frequency of TE–gene chimeric transcripts. There was a distinct inverse correlation between changes in the gene expression level and the relative frequency of TE-gene chimeras, whereupon downregulated genes were found with an elevated chimerism, and upregulated genes with a reduced chimerism. Statistical analysis showed that there was a strong and highly significant negative correlation between the rate of change in expression and chimeric frequency (Pearson: r = −0.556, *p* = 4.69 × 10^−119^; Spearman: r = −0.601, *p* = 7.34 × 10^−144^). The inverse correlation observed implies an association between increased transcription of TE-associated chimeric transcripts and genes experiencing transcriptional repression. The treatment-related disruption of transcription or RNA processing may promote the use of cryptic TE-derived splice or initiation sites, particularly when normal gene expression is reduced. Nevertheless, these findings represent correlations rather than definitive mechanistic pathways. It is important that genes like *LOC124902175*, *H4C15*, and *PHKB* showed significant increases in chimeric events even though they were downregulated. On the other hand, genes like *LRRC7*, *SLC2A9*, and *JPH2* showed the opposite behavior. These results suggest that transcriptional repression can promote greater TE exonization and chimeric transcript output, further pointing to a mechanistic connection between expression and TE activities (Figure 7A). To assess potential regional enrichment, we examined where TE–gene chimeric events were located on the chromosomes. The treated samples showed more chimeric events on most chromosomes. The largest increases were seen on chromosomes 1, 7, 11, 12, 17, and 19. In contrast, chromosomes 13, 14, 15, and 21 showed little change. Despite these localized differences, overall chromosomal distribution was not significantly altered by treatment (χ^2^ = 2.08, d.f. = 23, *p* = 1.00; Mann–Whitney U = 221.5, *p* = 0.173) (Figure S10). The results suggest that while some chromosomes show increased chimeric activity, the overall distribution of TE–gene chimeras across the genome remains largely unchanged, regardless of the conditions. To further contextualize exonized events, we performed epigenetic overlap analysis and observed 10,393 intersections between exonized chimeras and candidate cis-regulatory elements (cCREs). This enrichment suggests that a substantial fraction of TE exonization occurs within regulatory-active genomic regions, potentially altering local chromatin accessibility and transcription factor binding landscapes (Figure 7B).

### 2.6. GO and KEGG Pathway Analysis of Genes Involved in Chimeric Transcript Formation

**Figure 8:**
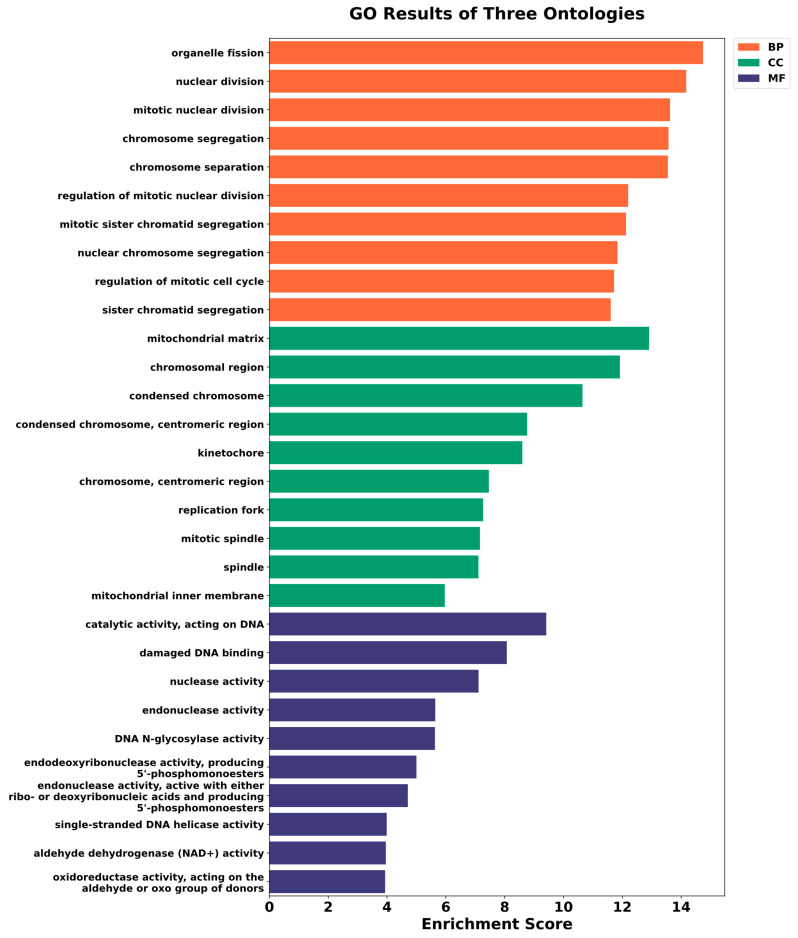
GO analysis of genes associated with upregulated TEs chimeras. The *y*-axis shows the three ontology processes, and the *x*-axis shows the scores for enrichment.

We performed GO enrichment analysis for genes associated with TE-gene chimeric transcripts following combined 125I and carfilzomib treatment. Upregulated genes were mainly enriched in processes related to cell division, including nuclear and mitotic division, along with functions linked to DNA activity and damage response (Figure 8). These genes were also associated with chromosomal regions and mitochondrial components. Conversely, genes that were downregulated were mostly linked to processes related to autophagy, including macroautophagy and the organization of autophagosomes, as well as related cellular structures like the autophagosome and phagophore assembly site (Figure S11). At the molecular level, these genes showed reduced enrichment in enzymatic activity related to lipids and functions involving RNA binding. Therefore, these findings indicate that combined genotoxic and proteotoxic stress promotes cell cycle progression and DNA repair while suppressing autophagy-related pathways.

The KEGG analysis of upregulated genes showed that these genes are involved in DNA replication and cell cycle control, base excision repair, Fanconi anemia, and p53 signaling pathways. The analysis identified supplementary metabolic pathways encompassing O-glycan biosynthesis, cofactor biosynthesis, ubiquinone and terpenoid-quinone biosynthesis, selenocompound metabolism, and GPI-anchor biosynthesis. The results show that the genes that are upregulated are important for cell growth, protecting the genome, and basic metabolic processes (Figures S12 and S13).

## 3. Discussion

Our results demonstrate that combined treatment with 125I and carfilzomib altered the landscape of TE-gene chimeric transcripts in KYSE-150 cells, resulting in both qualitative and quantitative changes in the cellular transcriptomic architecture. Additionally, our results indicate a multi-layered remodeling process that includes the transcription of canonical transposable elements as well as the formation of TE-gene chimeras, indicating coordinated but partially different regulatory responses to therapeutic stress. Combined genotoxic and proteotoxic stress differentially influenced the formation of TE-gene chimeric transcripts, as evidenced by an increase in TE-initiated and TE-exonic types, but a decrease in TE-terminal events. The observed treatment effects align with previous research, which has demonstrated that cellular stressors, including radiation and proteasome inhibition, modify the transcription and RNA processing of transposable elements [33,34,35]. 

At the TE family level, our data highlight that LINE, LTR, and MER elements were major contributors to increased TE-initiated and intronic chimeras following treatment, while embedded-type associations declined. The observed results align with previous studies, which have demonstrated that LINE and LTR elements exhibit high sensitivity to stress-induced chromatin remodelling and transcriptional deregulation [36,37]. Notably, ERV1 LTR elements showed significant deregulation in TE transcription analysis, while SINE/Alu families were the main drivers of TE-gene chimeras. This suggests that chimera remodeling may involve promoter capture and selective exonization rather than being a passive reflection of global TE activation. In contrast, the treatment seems to target specific intragenic TE-gene interactions by changing splicing accuracy or making intronic TE insertions less accessible. The expansion of both TE and gene partners across chimeric categories supports the concept that stress promotes transcriptional diversification through TE engagement. The greater number of unique TEs and genes in treated cells suggests that TE activity under stress is not limited to the amplification of pre-existing events, but also extends to the recruitment of novel partners. This is in agreement with studies showing that TE-gene chimeric transcripts can emerge as part of adaptive transcriptional programs in response to cellular stressors [38,39,40]. Importantly, although exonized chimeras expanded in number, the majority of core TE-gene associations were retained across conditions, indicating that stress enhances transcriptomic complexity without merely disrupting established splicing networks.

Analysis of partner diversity revealed that specific TE families, such as AluSx, AluJb, and MIRb, exhibited broad interaction capacity, consistent with their known abundance and propensity to generate splice acceptor and donor sites [41,42,43]. Conversely, the restricted gene partner usage observed for certain lncRNAs (e.g., *LINC00174*, *LINC01000*) indicates gene-specific biases in TE incorporation, which has been similarly noted in other cancer transcriptome studies [44,45]. The extensive exonization capacity of Alu and MIR families is particularly notable, given prior evidence that these elements contribute to alternative splicing and isoform diversification in human tissues [46]. Together, these findings reinforce the idea that SINE elements function as stress-responsive fusion hubs capable of rapidly expanding transcript diversity.

Differential expression analysis of TEs further supports the notion that combined treatment exerts selective pressure on TE activity. Although global TE transcript numbers did not markedly change, 43 elements were significantly altered across multiple families, including HERVs, LINEs, and Alus. This selective regulation mirrors previous findings that stress responses often result in family and locus-specific modulation rather than global activation of TEs [47,48,49]. Importantly, even low-abundance TEs that exhibit significant fold changes may disproportionately affect transcriptional networks, either by producing novel chimeric transcripts or by disrupting local chromatin states.

The association between TE chimeric transcript formation and changes in gene expression provides further mechanistic insight. Several genes, including *CEACAM19*, *ARB2BP*, *IL1RL1*, *RSAD2*, and *SPANXN1*, exhibited inverse relationships between their canonical expression and the emergence of TE-derived isoforms. These data support a model in which TE-driven transcriptional initiation, exonization, or termination can disrupt associated gene expression, as previous studies have demonstrated that TE insertions disrupt or reduce canonical gene transcription [50,51]. The genes *OLFML2A* and *D2HGDH* showed increased expression levels even though their TE-derived isoforms underwent significant rearrangements, which indicates that TE chimerism can occur with either gene suppression or activation based on genomic and regulatory factors. High-confidence candidates such as *SPANXN1*, *IL1RL1*, and *RSAD2* were further given priority by our functional impact scoring, indicating that these TE-associated events may represent biologically relevant candidates for future investigation of stress-responsive immune and regulatory pathways.

Beyond their influence on gene regulation, chimeric transcripts originating from TEs could also elicit immunogenic responses. The integration of TE sequences has the potential to produce novel peptide products that may function as tumor-specific neoantigens and subsequently be presented via MHC class I pathways [52,53]. Consequently, aberrant transcription of TEs could potentially trigger innate immune responses via nucleic acid-sensing pathways [54,55]. Although these particular aspects were not directly examined in this investigation, our results suggest a potential link between stress-induced TE activity and tumor immunogenicity in the context of ESCC.

We found a clear inverse relationship between changes in associated-gene expression and TE chimerism: genes that lost expression tended to show higher rates of TE exonization (Pearson r = −0.556, *p* = 4.69 × 10^−119^; Spearman ρ = −0.601, *p* = 7.34 × 10^−144^). This pattern echoes prior observations in cancer transcriptomes and could reflect more than one process in our system. For instance, less production of canonical transcripts could reveal hidden splice sites in nearby TEs, or TE transcription that is now active could get in the way of normal gene expression. The data support both scenarios, and they are not mutually exclusive. Furthermore, the hypothesis that stress-induced TE incorporation preferentially takes place within regulatory-active chromatin domains was supported by the extensive overlap between exonized chimeras and candidate cis-regulatory elements. The joint treatment of 125I and carfilzomib altered the TE-gene chimeric transcriptome by enhancing TE initiation and exonization, along with partner diversity, while preserving the inverse relationship between TE activity and associated gene expression. These results back up the idea that TEs are flexible regulators of gene expression when cells are under stress. This suggests that stress-induced chimeras may help the cancer cell transcriptome adapt.

This study has several limitations. First, our analyses rely on short-read RNA sequencing, which limits accurate reconstruction of full-length TE–gene chimeric isoforms; long-read transcriptomic approaches would provide improved structural resolution. Second, although we observed strong statistical associations between TE incorporation and changes in host gene expression, these findings remain correlative, and direct causal relationships were not experimentally validated and will require targeted functional assays. Third, our results are derived from a single esophageal cancer cell line (KYSE150), which limits the generalizability of our findings; validation in additional ESCC models and primary tumor samples will be necessary to assess broader applicability. In addition, as with other transcriptome-wide analyses of repetitive elements, the identification of differentially expressed TEs and TE-gene chimeric events depends on computational inference and threshold selection, and some marginal events may be sensitive to normalization and statistical cutoffs. Future studies integrating long-read sequencing, chromatin accessibility profiling, and functional perturbation approaches will be important to further elucidate the mechanistic role of TE–gene chimeras in cancer.

## 4. Materials and Methods

### 4.1. Data Retrieval and Preprocessing

We obtained RNA-seq data from the NCBI Sequence Read Archive (SRA) for KYSE150 esophageal squamous carcinoma cells that had been treated with both 125I radiation and carfilzomib, as well as untreated control samples (three biological replicates per condition). The accession numbers for these were SRR29055194, SRR29055195, SRR29055196, SRR29055197, SRR29055198, and SRR29055199. The corresponding sample grouping was as follows: SRR29055194, SRR29055195, and SRR29055196 represent KYSE150 cells treated with combined 125I radiation and carfilzomib, whereas SRR29055197, SRR29055198, and SRR29055199 correspond to untreated control replicates. FASTQ files were obtained using the SRA Toolkit (https://github.com/ncbi/sra-tools, accessed on 16 October 2025) and fastq-dl (https://github.com/rpetit3/fastq-dl, accessed on 18 October 2025). Data quality was assessed using FastQC (https://github.com/s-andrews/FastQC, accessed on 2 November 2025). Adapter trimming and removal of low-quality bases were performed using Trimmomatic v0.39 [56] with default parameters. Reads with Phred quality scores < 20 and lengths < 36 bp after trimming were discarded. The resulting high-quality reads were used for downstream analyses.

### 4.2. Characterization of Differentially Expressed TEs

We utilized preprocessed, high-quality RNA-seq FASTQ files from two experimental conditions (treatment and control), each comprising three biological replicates, to conduct differential expression analysis of TEs using the TEtranscripts v2.2.3 software [57]. Before quantification, we created aligned BAM files by mapping the RNA-seq reads to the human reference genome (GRCh38) using the STAR v2.7.11b aligner [58]. We followed the specific mapping parameters suggested in the TEtranscripts documentation to make sure that multi-mapped reads from repetitive TE sequences were handled correctly. These BAM files, along with the corresponding GTF annotation files for genes and TEs, served as input to TEtranscripts. The software jointly quantified gene and TE expression and performed normalization and differential expression analysis using the DESeq2 v1.44.x [59] statistical framework. TEtranscripts produced differential expression results at both the individual TE family and TE subfamily levels, encompassing log2 fold changes (log2FC) and adjusted *p*-values (padj). For subsequent visualization, we utilized customized R scripts to produce volcano plots, MA plots, and heatmaps, facilitating a thorough depiction of TE expression variations between treatment and control conditions.

### 4.3. Characterization of TE-Gene Chimeric Transcripts in KYSE150 Cells

The preprocessed RNA-seq data were analyzed to identify TE-gene chimeric transcripts using ChimeraTE v1.0 software [60]. ChimeraTE has two modes of operation: Mode 1, which needs a reference genome, and Mode 2, which does not. The most recent human reference genome (GRCh38.p14, NCBI accession: GCF_000001405.40) was used in the present work with Mode 1. The RepeatMasker BED file was changed to GTF format, which is what ChimeraTE needed, and the gene GTF annotation file was downloaded. After specifying the full paths of FASTQ files for three biological replicates in the input.txt configuration file, the software was executed on a Linux platform. The analysis yielded three output files (TE_initiated.tsv, TE_exonic.tsv, and TE_terminal.tsv), which were subsequently used for visualization and downstream analysis.

### 4.4. Motif Prediction and Analysis of Chimeric Transcript-Associated Genes

Differentially expressed TEs contributing to chimeric transcript formation were extracted in FASTA format using a locally developed Python script (can be accessed at https://github.com/majie5976/TE_chimera_analysis). Motif prediction was performed with FIMO v5.5.x (from the MEME Suite [61]) using the JASPAR [62] vertebrate non-redundant database, and results were visualized with an in-house Python script. In parallel, FASTA sequences of genes involved in TE-gene chimeric transcripts were extracted. To evaluate the influence of TEs on gene expression, gene quantification was performed with Salmon [63] (https://github.com/COMBINE-lab/salmon, accessed on 7 January 2026), followed by differential expression analysis using DESeq2. Visualization of expression patterns was carried out with custom R v3.6.3 scripts (https://github.com/majie5976/TE_chimera_analysis).

### 4.5. Epigenetic Context and Regulatory Element Overlap Analysis

To investigate the regulatory potential of TE insertions, we performed genomic overlap analysis with candidate cis-Regulatory Elements (cCREs). The candidate cis-regulatory element (cCRE) annotations were obtained from the ENCODE Project (GRCh38/hg38) in BED format. These include promoter-like, enhancer-like, and CTCF-only elements, which were grouped accordingly for downstream analysis. Overlaps between TE insertion coordinates and cCRE regions were calculated using BEDTools v2.30.0 [64]. Overlapping regions were classified by cCRE category (promoter-like, enhancer-like, and CTCF-only). Statistical enrichment of overlaps was assessed using Fisher’s exact test by comparing observed overlaps to background genomic expectations.

### 4.6. Functional Enrichment Analysis of Chimeric Genes

Genes linked to TE-gene chimeric transcripts underwent Gene Ontology (GO) and Kyoto Encyclopedia of Genes and Genomes (KEGG) pathway enrichment analysis. We used the clusterProfiler v4.18.4 R package [65] to do an enrichment analysis to make sure the results were statistically reproducible and clear. We used the Benjamini–Hochberg method to calculate padj, and pathways with an FDR of less than 0.05 were considered to be significantly enriched.

### 4.7. Functional Impact Scoring and Integrated Candidate Prioritization

To evaluate the functional importance of TE-gene chimeric events, we established a multi-parameter impact scoring system that incorporates various tiers of biological evidence. The composite functional impact score included log2 fold-change magnitude (40% weight), TE family impact potential based on known biological mechanisms (30% weight), epigenetic regulatory context (20% weight), and consistency across biological replicates (10% weight). We chose weighting parameters based on how relevant they were to biology and tested them for robustness through sensitivity analysis to make sure that the candidate ranking would stay the same. Candidates with composite scores higher than 0.8 were called high-impact candidates.

To rank candidates more thoroughly, we also used an integrated scoring framework that included differential expression significance (35%), functional impact score (25%), epigenetic context evidence (20%), pathway relevance (10%), and cross-analysis consistency (10%). We ranked genes based on their overall composite scores and chose the top 29 high-confidence candidates for more biological interpretation and downstream analysis. The Supplementary Materials include custom Python scripts that can be used to do these scoring and ranking tasks again.

## 5. Conclusions

In KYSE150 cells, 125I radiation and carfilzomib altered TE transcription and TE-gene chimeric transcript creation. This was characterized by increased TE-initiated and intronic exonization events, alongside a reduction in TE-terminal chimeras, accompanied by expanded TE and gene partner diversity. Chimera activity was higher on some gene-rich chromosomes, but genomic distribution remained the same, indicating organized transcriptome disruption. Gene expression levels showed a strong inverse correlation with chimerism frequency and TE incorporation. This does not prove causality, but it suggests that therapeutic stress may be associated with increased TE exonization and changes in transcriptional regulation, including altered splicing or gene expression. Pathway analysis indicated that genotoxic and proteotoxic stress were associated with cell cycle, DNA repair, and autophagy-related pathway activation and suppression, respectively. In esophageal cancer cells, therapeutic stress is associated with changes in TE-gene interactions, the emergence of novel transcript isoforms, and broader transcriptome remodeling. These findings support the view that transposable elements are dynamic components of the cancer stress response and provide a framework for future functional studies of their biological and therapeutic relevance. These findings also suggest that TE-derived isoforms may contribute to tumor immunogenicity, including the generation of neoantigens, and could be explored in future immunotherapy approaches in ESCC.

## Data Availability

The data used in this study can be accessed through the NCBI BioProject number PRJNA936875 or the SRR numbers: SRR29055194, SRR29055195, SRR29055196, SRR29055197, SRR29055198, and SRR29055199.

## Supplementary Materials

The following supporting information can be downloaded at: https://www.mdpi.com/article/10.3390/ijms27083471/s1.
