# Supplementary material for: Therapeutic Stress-Induced Remodeling of Transposable Elements and TE-Gene Chimeras in KYSE150 Esophageal Squamous Cell Carcinoma Cells
Source: Int J Mol Sci. 2026 Apr 13;27(8):3471. doi: 10.3390/ijms27083471 (PMC13116134; doi:10.3390/ijms27083471)
Supplement: Supplementary file 1 [file ijms-27-03471-s001.zip › Supplementary_Information.pdf]

# Supplementary Information

## Therapeutic Stress-Induced Remodeling of Transposable Elements and TE-Gene Chimeras in KYSE150 Esophageal Squamous Cell Carcinoma Cells

The comparison of TE family distribution between normal and treated samples shows that all TE families are consistently more abundant in normal samples. The Alu family is the most prevalent, with 8,038 counts in the normal group and 7,893 in the treated group. The other category follows, with 7,101 counts in the normal group and 7,014 in the treated group, while LINE elements account for 5,344 in the normal group and 5,254 in the treated group. The MER family shows 2,018 counts in the normal group and 1,944 in the treated group, and LTR elements are the least abundant, with 618 in the normal group and 605 in the treated group. Overall, although the magnitude of the difference varies across families, the data reveal a consistent trend in which TE counts are slightly reduced in treated samples compared to normal.

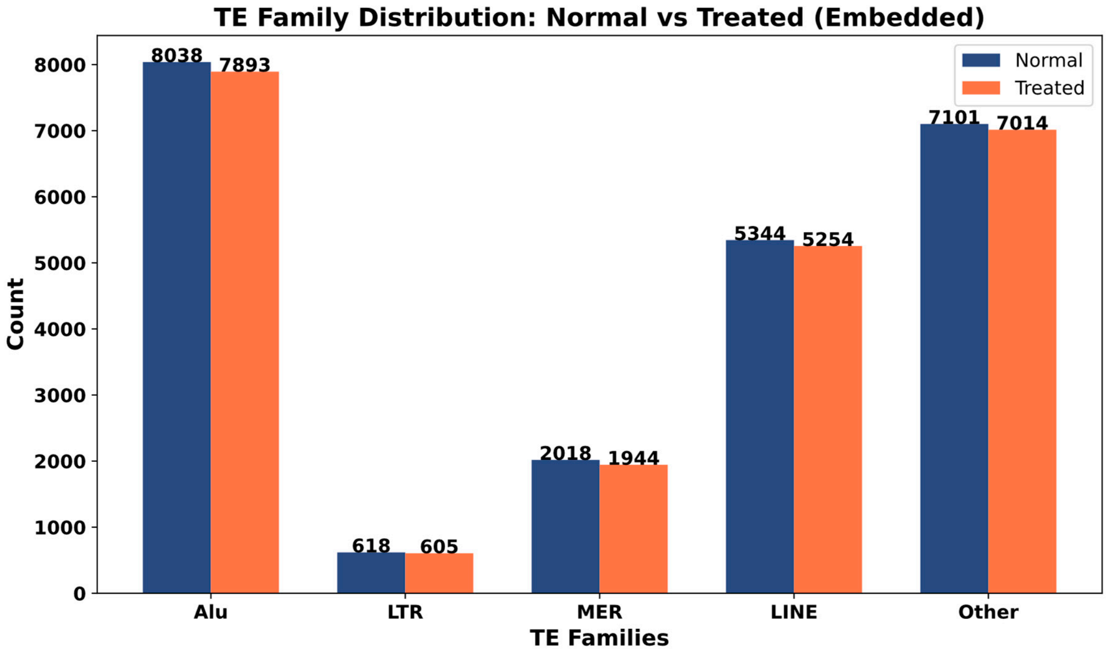


**Figure S1: Distribution of Transposable Element (TE) families between normal and treated samples for embedded type**. Bars represent the counts of each TE family, with normal shown in blue and treated in orange.

Across all TE families examined (Alu, LTR, MER, LINE, and Other), the treated group consistently displayed slightly higher counts compared to the normal group. Specifically, the Alu family increased from 700 in normal to 726 in treated samples, while LTR elements showed only a minor increase (from 126 to 130). The MER family rose from 199 in normal to 204 in treated, and LINE elements increased from 628 to 633. The largest absolute difference was observed in the other category, which increased from 803 in the normal samples to 847 in the treated samples. Overall, while differences across families were modest, the trend suggests a general increase in TE family counts in the treated condition compared to the normal condition.

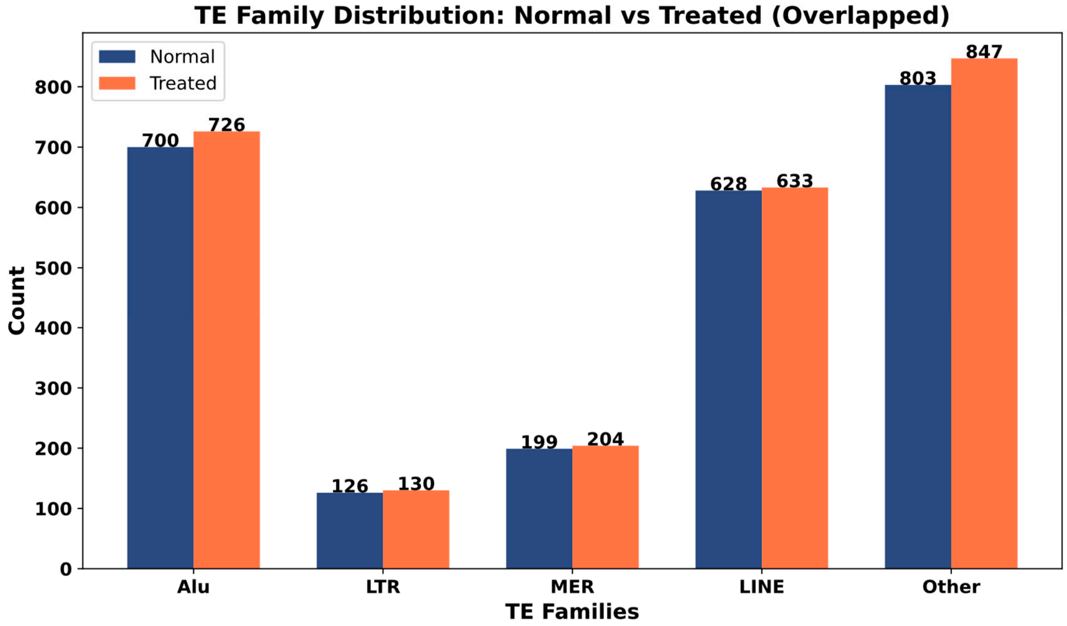


**Figure S2: Distribution of TE families between normal and treated samples for overlapped type**. Bars represent the counts of each TE family, with Normal shown in blue and treated in orange.

A significant increase in TE counts was observed across all families in the treated group compared to the untreated. The Alu family showed the largest difference, rising from 4247 in the normal group to 5281 in the treated samples. Similarly, LINE elements increased notably (2110 vs. 3209), as did the other category (2653 vs. 3702). More modest increases were noted in MER elements (583 vs. 827) and LTRs (199 vs. 266). Overall, intronic TE insertions remained consistently higher in the treated condition, with the most prominent differences observed in Alu, LINE, and other families.

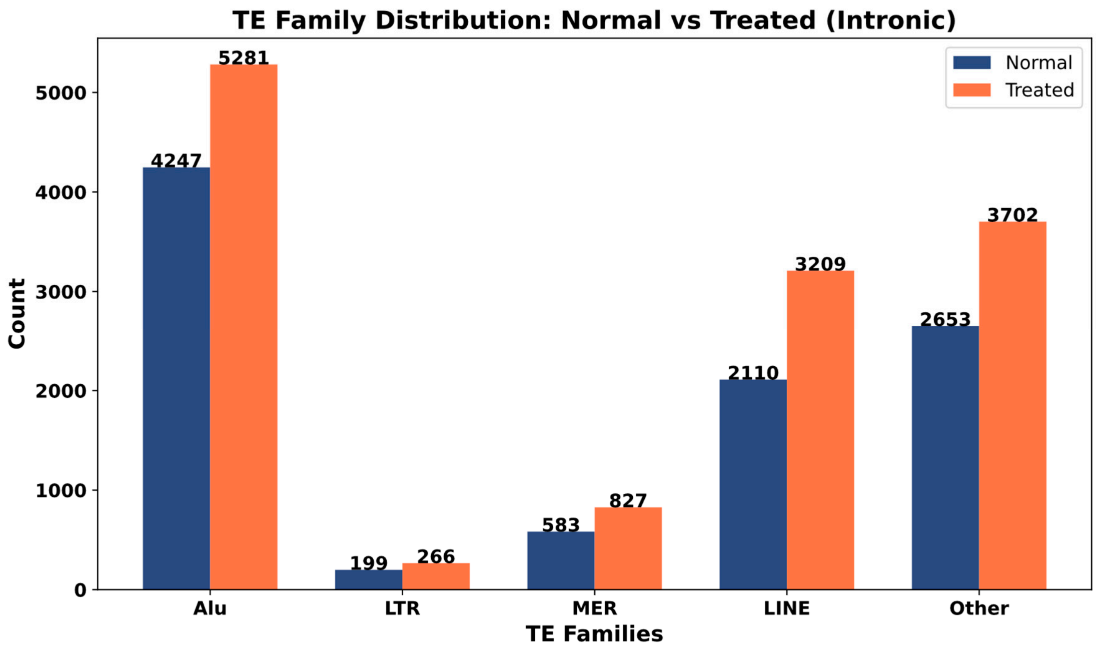


**Figure S3: Distribution of TE families between normal and treated samples for intronic type**. Bars represent the counts of each TE family, with Normal shown in blue and treated in orange.

In the TE-terminal (downstream) category, 112 DEGs were identified, of which 52 were upregulated and 60 were downregulated. We have plotted a heatmap of the top 50 up- and down-regulated genes associated with the formation of TE-gene chimeric transcripts.

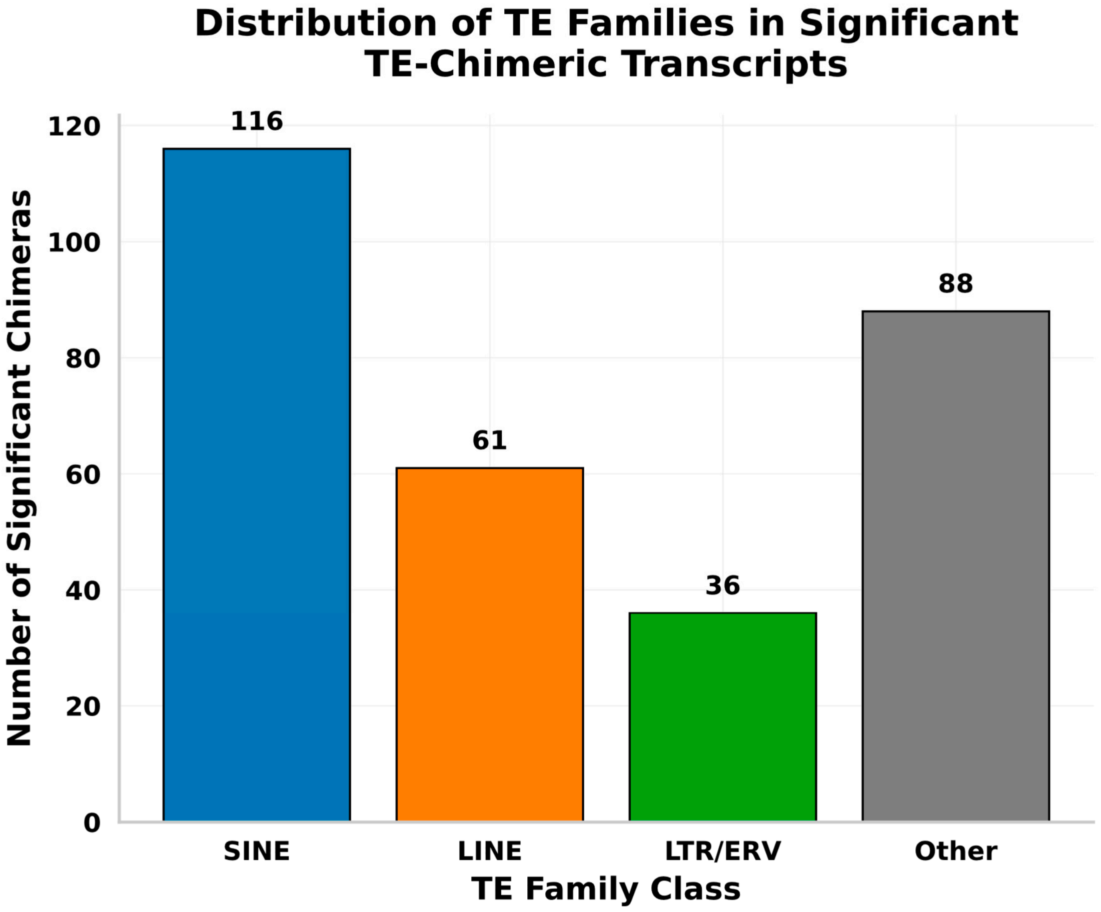


**Figure S4: Distribution of transposable element (TE) families contributing to significant TE-gene chimeric transcripts**. Bar plot showing the number of statistically significant TE-gene chimeras (FDR < 0.05) associated with individual TE families.

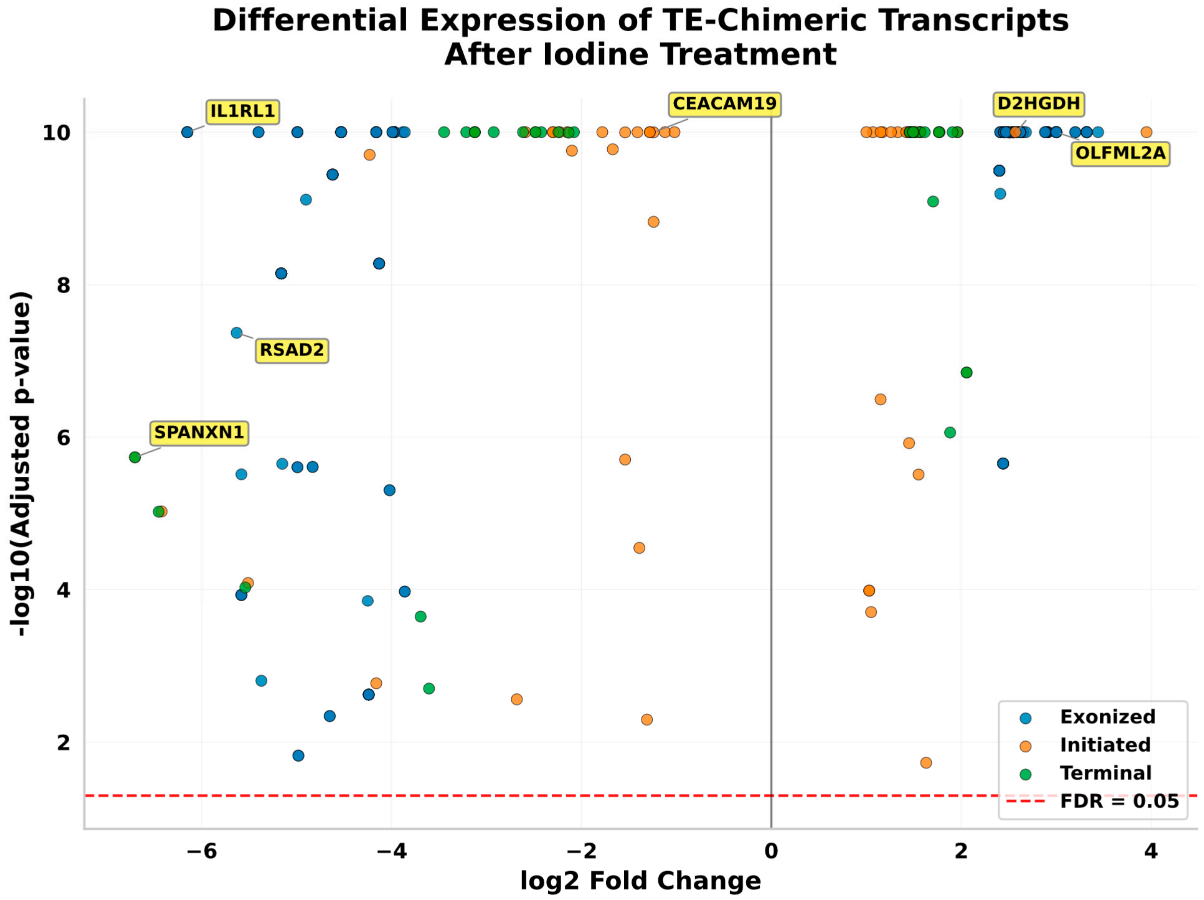


**Figure S5: Differential expression of TE-gene chimeric transcripts following iodine treatment.** Volcano plot displaying log2 fold change (treated vs. control) versus -log10 adjusted p-value for TE-gene chimeric transcripts. Points are colored according to chimera category (exonized, TE-initiated, and TE-terminal). The horizontal dashed red line indicates the significance threshold (FDR = 0.05).

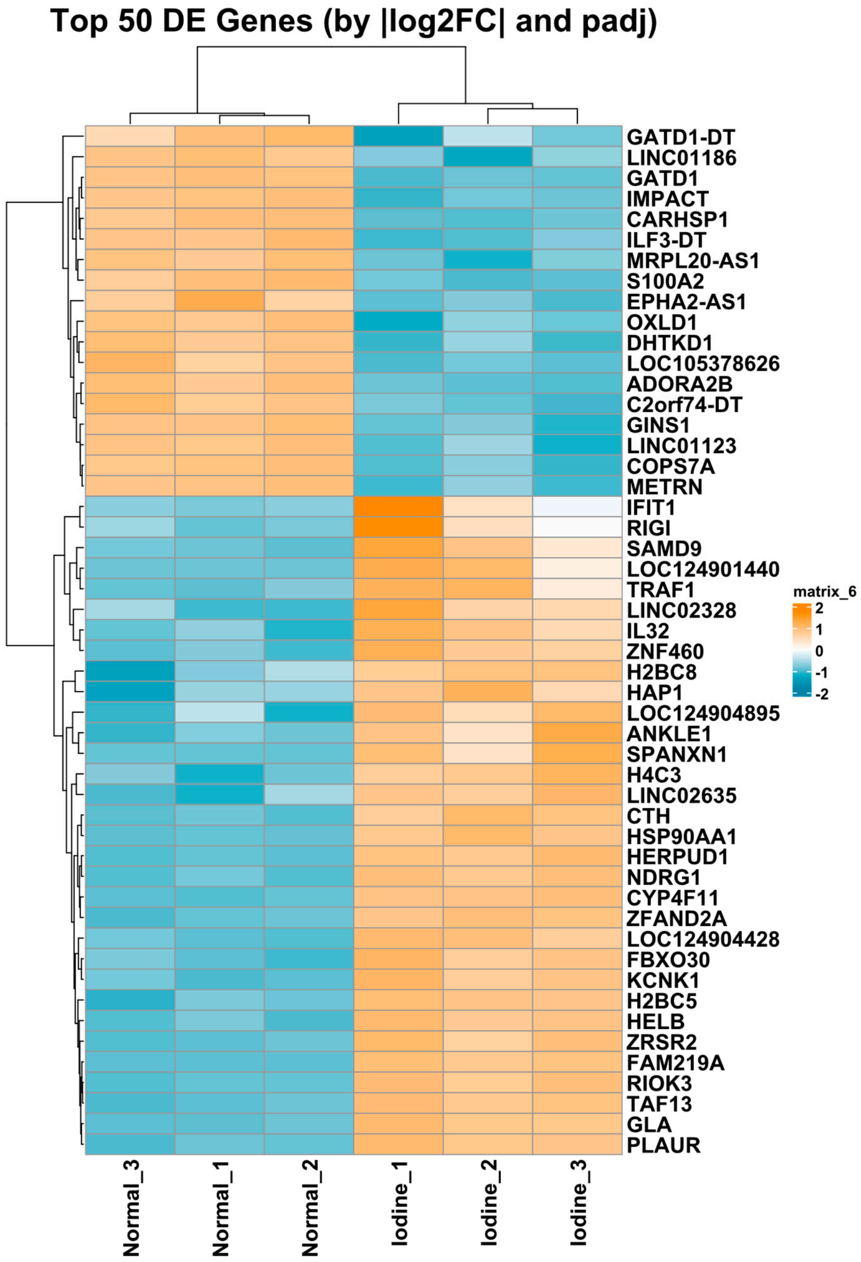


**Figure S6: DEGs associated with chimeric transcript formation after treatment.** The heatmap displays the top 50 DEGs associated with TE-terminal type of chimeric transcripts with respect to log2FC and p-adj values.

For TE-initiated transcripts, we observed 42 DEGs, evenly split between 21 upregulated and 21 downregulated. To visualize these patterns, we generated a heatmap of all the DEGs for the TE-initiated type, ranked according to log2fold change and adjusted p-value (p-adj).

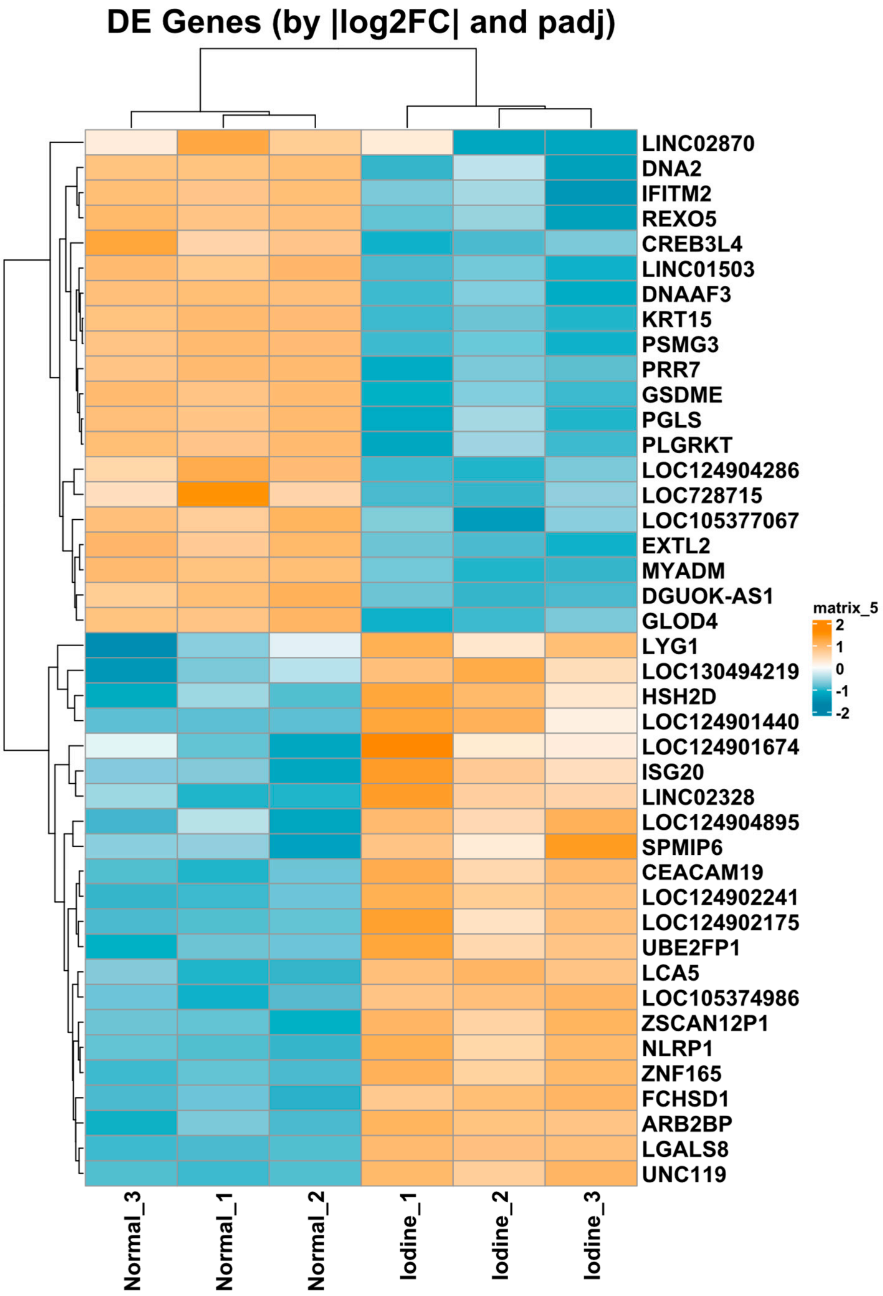


**Figure S7: DEGs associated with chimeric transcripts formation after treatment.** The heatmap displays the top 50 DEGs associated with TE-initiated type of chimeric transcripts with respect to log2FC and p-adj values.

We first examined the distribution of transcription factor binding motifs on TEs located in the upstream regions of genes. The heatmap of motif abundance (Figure, top panel) revealed that only a subset of TEs harbored a high density of motifs, with Alu subfamilies (AluJb, AluSc8, AluSz) and L1 elements (L1ME3Cz, L1PA12) showing the strongest enrichment. Among the identified motifs, members of the zinc finger (ZNF) family (e.g., ZNF135, ZNF460, ZNF140, ZNF281, ZNF558, ZNF740, and ZNF8), as well as KLF family factors (KLF3, KLF5, KLF17), were particularly abundant across multiple TEs. Other motifs such as PRDM9, POU1F1/POU2F2, and FOXC2 were detected at lower but notable frequencies. This suggests that specific TE subfamilies may provide regulatory input to upstream regions by binding transcription factors in a recurrent manner.

To further investigate motif relationships, we assessed pairwise correlations among the most frequent motifs (Figure, bottom panel). Strong positive correlations were observed among KLF3, KLF5, and KLF17, suggesting that these motifs often co-occur within the same TEs, potentially reflecting coordinated regulation by KLF family transcription factors. Several ZNF motifs also displayed moderate correlations with each other, consistent with overlapping or redundant DNA-binding preferences within this protein family. In contrast, specific motifs (e.g., PRDM9, NR3C2) showed weaker or negative correlations, suggesting distinct motif usage patterns across TEs. Together, these analyses indicate that upstream TEs serve as hubs for transcription factor binding, particularly enriching for KLF and ZNF motifs, and that these motifs often occur in correlated patterns, potentially influencing gene regulation under specific conditions.

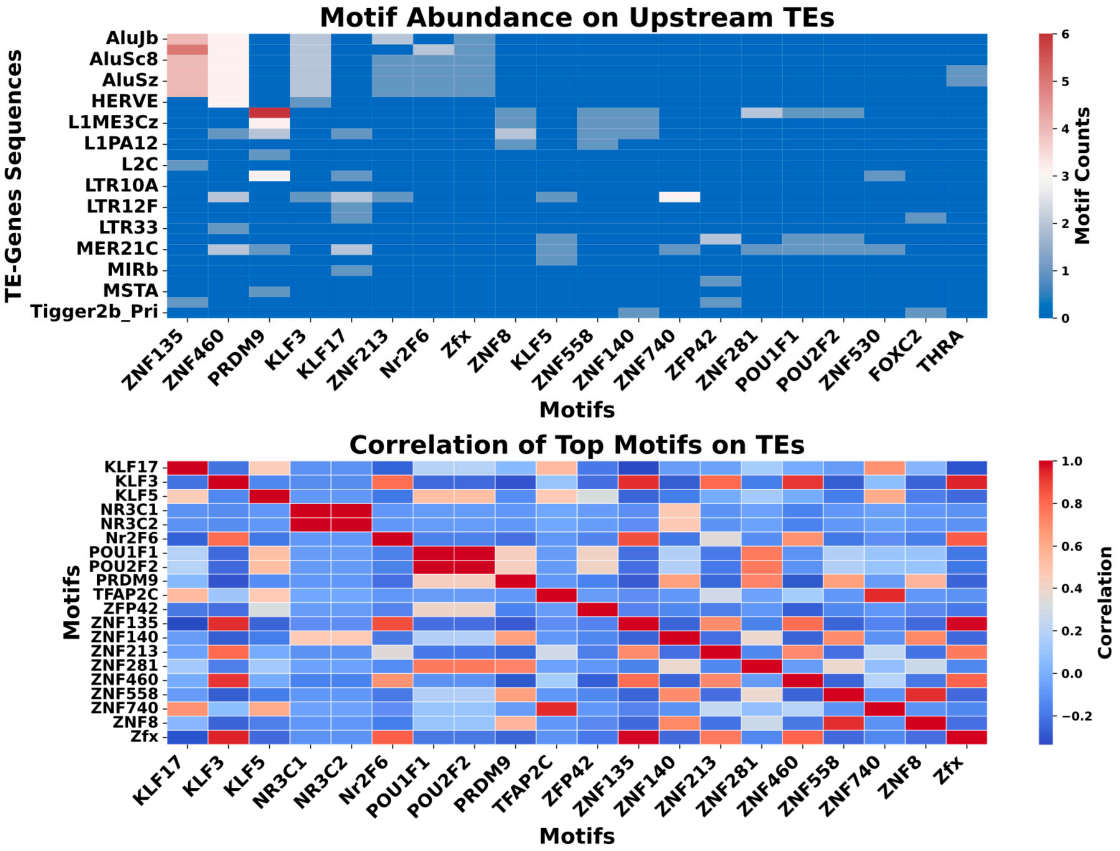


**Figure S8: Motif prediction on TEs present in the upstream region of the genes associated with chimeric transcript formation.**

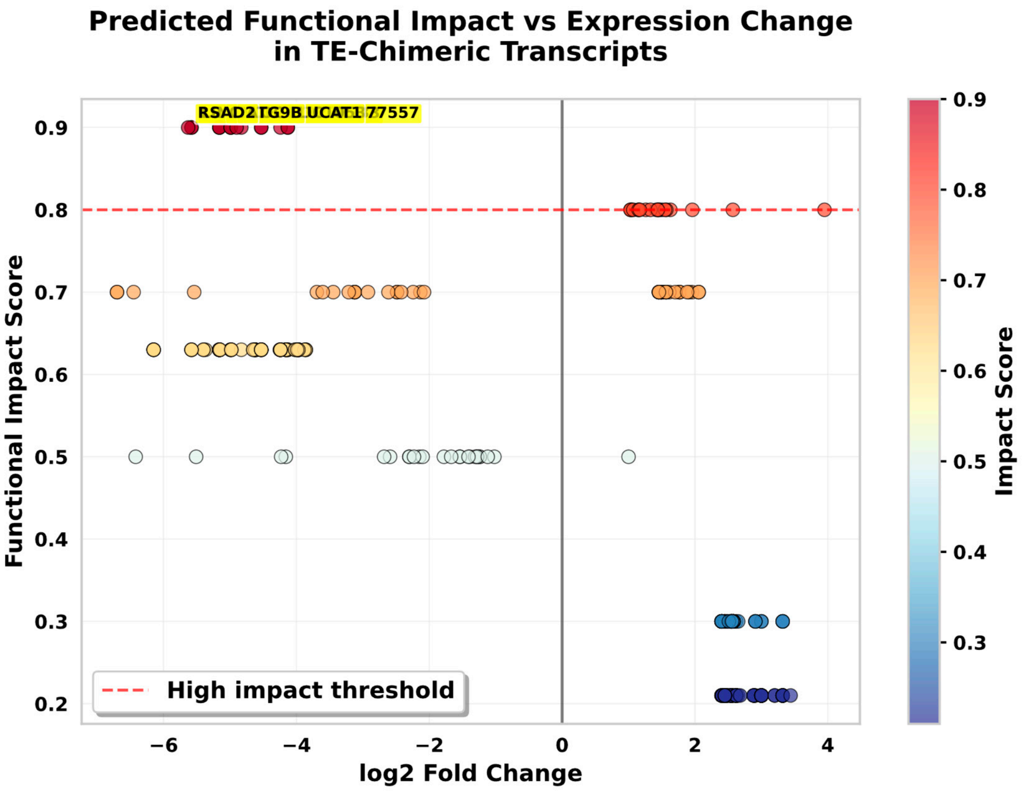


**Figure S9: Relationship between predicted functional impact and expression change in TE-gene chimeric transcripts.** Scatter plot illustrating the association between log2 fold change (treated vs. control) and composite functional impact score for TE-gene chimeric events. The dashed red line (0.8) indicates the high-impact threshold. High-impact candidates.

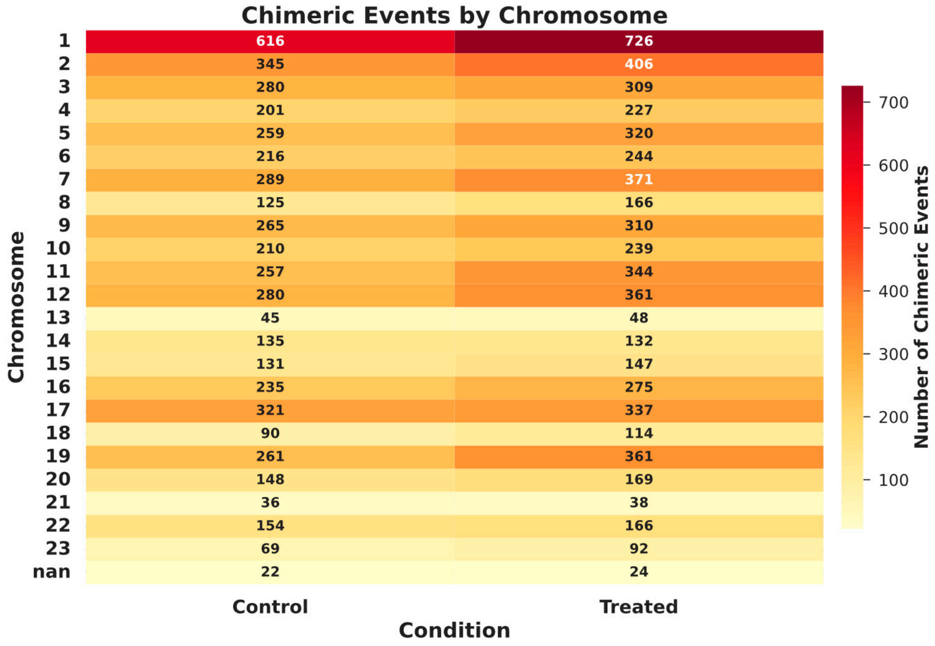


**Figure S10: Chromosomal localization of TE-gene chimeric events.** Chromosomal localization of TE-gene chimeric events in untreated and treated samples with iodine. Heatmap of the number of chimeric events at each chromosome across conditions.

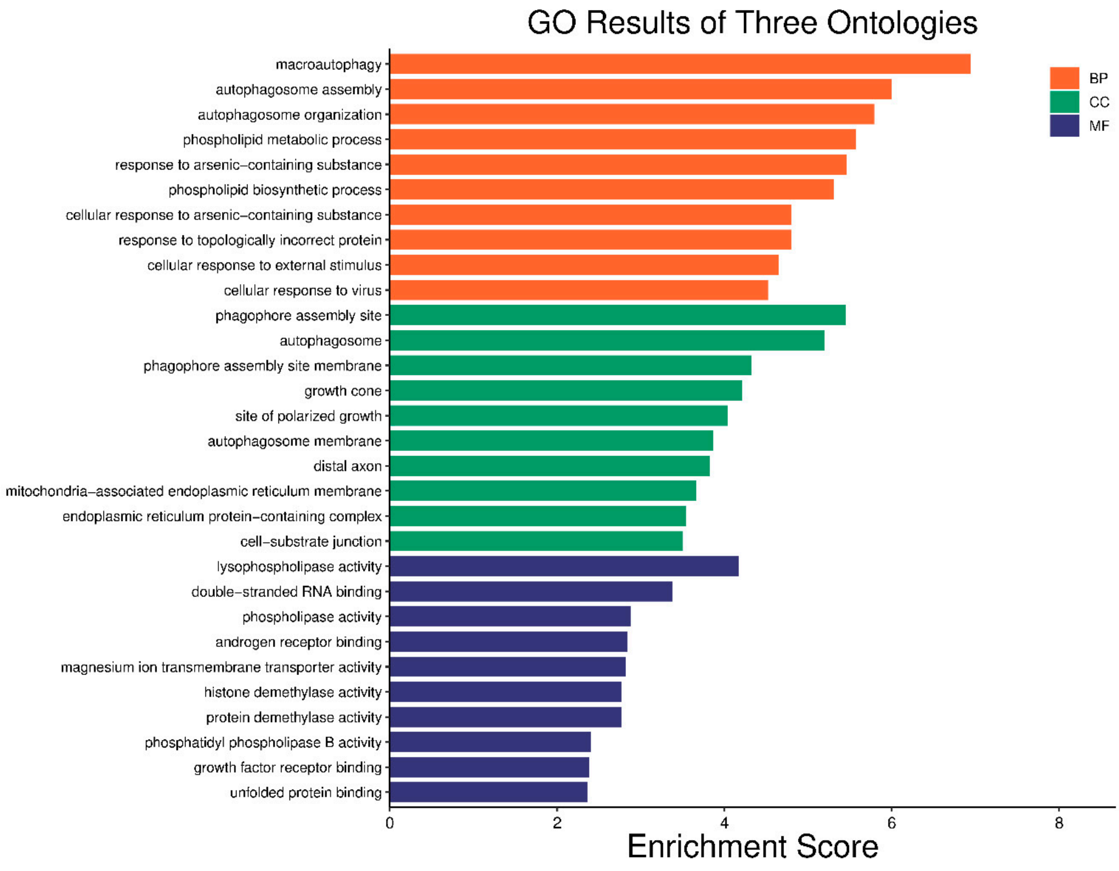


**Figure S11: GO analysis of upregulated TEs chimeric-associated genes**. The y-axis shows the three ontology processes, and the x-axis displays enrichment scores.

## KEGG Pathway Enrichment Analysis

KEGG pathway analysis revealed significant enrichment in pathways primarily associated with DNA maintenance, cell cycle regulation, and stress response. Among the top enriched pathways were DNA replication, cell cycle, base excision repair, Fanconi anemia pathway, and the p53 signaling pathway, all of which are closely related to genomic stability and cell proliferation control. In addition, several metabolic and biosynthetic pathways, including other types of O-glycan biosynthesis, biosynthesis of cofactors, ubiquinone and other terpenoid–quinone biosynthesis, selen compound metabolism, and glycosylphosphatidylinositol (GPI)-anchor biosynthesis, were significantly overrepresented. The enrichment scores (–log10 p-value) indicated strong statistical significance ($p < 0.001$), with the DNA replication and cell cycle

pathways showing the highest enrichment values. These findings suggest that the differentially expressed genes are predominantly involved in cell cycle regulation, DNA repair mechanisms, and metabolic processes.

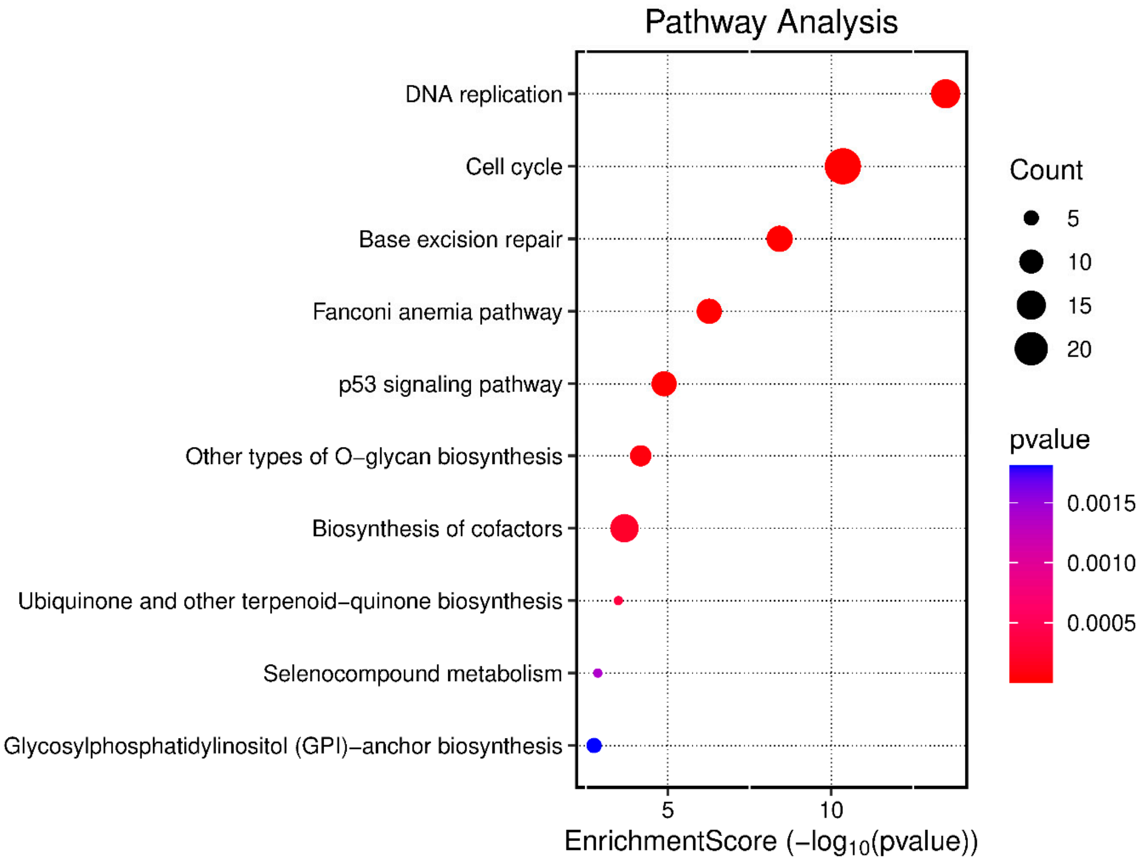


**Figure S12: KEGG pathway analysis of upregulated TEs chimeric-associated genes**. The y-axis represents pathways and processes, while the x-axis displays enrichment scores.

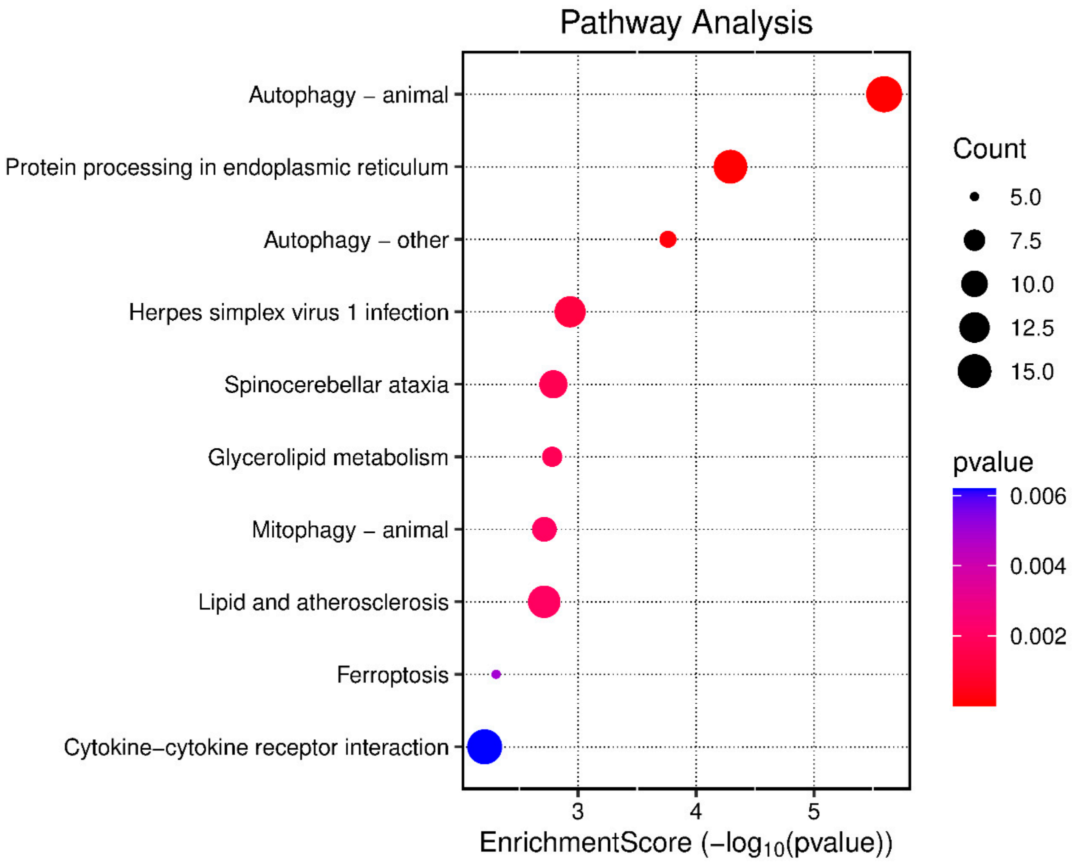


**Figure S13: KEGG pathway analysis of downregulated TEs chimeric-associated genes**. The y-axis represents pathways and processes, while the x-axis displays enrichment scores.

**Table S1: Chimeric transcripts detected between genes and transposable elements (TE-initiated type).** Each row lists the gene identifier, strand orientation, and genomic position of the gene, along with the associated TE identifier, strand, and genomic coordinates. Columns report the number of chimeric reads detected in control versus iodine-treated samples, the cell condition where the chimera was observed (Normal or Treated), and differential expression statistics (log2 fold change and adjusted p-value) for the corresponding gene-TE junction.

| gene_id | gene_strand | gene_position | TE_id | TE_strand | TE_position | Chimeric_reads_control | Chimeric_reads_iodine | Normal Cells | Hypoxia | log2FoldChange | padj |
|---|---|---|---|---|---|---|---|---|---|---|---|
| **ARB2BP** | + | NC_000003.12:101515867-101518867 | L2a | - | NC_000003.12:101518506-101518728 | 0 | 8 | No | Yes | -1.78 | 6.35E-17 |
| **CEACAM19** | + | NC_000019.10:44662860-44665860 | MLT1L | - | NC_000019.10:44665717-44665860 | 0 | 21 | No | Yes | -1.24 | 4.94E-13 |
| **CREB3L4** | + | NC_000001.11:153964487-153967487 | Tigger19b | + | NC_000001.11:153966052-153966261 | 0 | 1 | No | Yes | 1.15 | 3.20E-07 |
| **DGUOK-AS1** | - | NC_000002.12:73981439-73984439 | L2c | - | NC_000002.12:73982061-73982404 | 0 | 2 | No | Yes | 1.55 | 3.10E-06 |
| **DNA2** | - | NC_000010.11:68472521-68475521 | AluSx | + | NC_000010.11:68472521-68472731 | 0 | 1 | No | Yes | 1.17 | 1.46E-14 |
| **DNAAF3** | - | NC_000019.10:55166722-55169722 | AluSx | + | NC_000019.10:55167346-55167649 | 0 | 2 | No | Yes | 1.44 | 2.02E-14 |
| **EXTL2** | - | NC_000001.11:100895179-100898179 | AluJb | + | NC_000001.11:100895397-100895552 | 0 | 1 | No | Yes | 1.07 | 5.18E-29 |

| | | | | | | | | | | | |
|---|---|---|---|---|---|---|---|---|---|---|---|
| **FCHSD1** | - | NC_000005.10:141651418-141654418 | L2a | + | NC_000005.10:141652107-141652881 | 0 | 1 | No | Yes | -2.15 | 5.05E-15 |
| **GLOD4** | - | NC_000017.11:785895-788895 | L1ME3G | + | NC_000017.11:785895-786095 | 5 | 14 | Yes | Yes | 1.15 | 7.89E-35 |
| **GSDME** | - | NC_000007.14:24795539-24798539 | LTR2 | - | NC_000007.14:24795539-24795789 | 1 | 2 | Yes | Yes | 1.57 | 5.56E-50 |
| **HSH2D** | + | NC_000019.10:16131028-16134028 | AluSz | + | NC_000019.10:16133800-16133913 | 0 | 3 | No | Yes | -1.24 | 1.40E-09 |
| **IFITM2** | + | NC_000011.10:304816-307816 | AluY | - | NC_000011.10:307492-307788 | 0 | 2 | No | Yes | 1.00 | 2.03E-15 |
| **ISG20** | + | NC_000015.10:88632632-88635632 | Tigger2b_Pri | - | NC_000015.10:88634948-88635632 | 0 | 1 | No | Yes | -2.10 | 7.50E-11 |
| **KRT15** | - | NC_000017.11:41518890-41521890 | MIRc | + | NC_000017.11:41521636-41521806 | 0 | 1 | No | Yes | 3.95 | 2.27E-191 |
| **LCA5** | - | NC_000006.12:79538782-79541782 | MIRb | - | NC_000006.12:79538782-79538973 | 0 | 1 | No | Yes | -1.41 | 1.51E-13 |
| **LGALS8** | + | NC_000001.11:236515214-236518214 | AluSx | - | NC_000001.11:236517652-236517969 | 0 | 2 | No | Yes | -1.12 | 3.65E-71 |
| **LINC01503** | + | NC_000009.12:129333923-129336923 | L2b | + | NC_000009.12:129333714-129334014 | 0 | 1 | No | Yes | 2.57 | 2.90E-33 |
| **LINC02328** | + | NC_000014.9:85931678-85934678 | LTR12F | + | NC_000014.9:85934518-85934678 | 0 | 1 | No | Yes | -5.51 | 8.20E-05 |
| **LINC02870** | + | NC_000010.11:132442189-132445189 | MER5A | - | NC_000010.11:132444483-132444548 | 0 | 2 | No | Yes | 1.63 | 0.018620176 |

| | | | | | | | | | | | |
|---|---|---|---|---|---|---|---|---|---|---|---|
| **LOC105374986** | + | NC_000006.12:26254500-26257500 | LTR22A | + | NC_000006.12:26257219-26257500 | 0 | 2 | No | Yes | -4.23 | 9.85E-11 |
| **LOC105377067** | + | NC_000003.12:46160474-46163474 | L1PA12 | - | NC_000003.12:46161737-46163474 | 0 | 1 | No | Yes | 1.45 | 1.20E-06 |
| **LOC124901440** | + | NC_000006.12:152835392-152838392 | LTR12C | + | NC_000006.12:152834160-152835658 | 0 | 3 | No | Yes | -6.42 | 9.45E-06 |
| **LOC124901674** | - | NC_000007.14:76164388-76167388 | AluSx | + | NC_000007.14:76164388-76164647 | 0 | 3 | No | Yes | -1.31 | 0.005088991 |
| **LOC124902175** | - | NC_000009.12:69171597-69174597 | AluSc | - | NC_000009.12:69171790-69172093 | 0 | 2 | No | Yes | -2.30 | 1.12E-16 |
| **LOC124902175** | - | NC_000009.12:69171597-69174597 | MER3 | + | NC_000009.12:69172339-69172550 | 0 | 2 | No | Yes | -2.30 | 1.12E-16 |
| **LOC124902241** | + | NC_000009.12:107337207-107340207 | AluSx | + | NC_000009.12:107340001-107340207 | 0 | 3 | No | Yes | -1.54 | 1.97E-06 |
| **LOC124904286** | + | NC_000018.10:36188122-36191122 | MSTA | + | NC_000018.10:36191047-36191122 | 3 | 0 | Yes | No | 1.03 | 0.000103233 |
| **LOC124904286** | + | NC_000018.10:36188122-36191122 | Tigger10 | - | NC_000018.10:36189717-36189822 | 0 | 1 | No | Yes | 1.03 | 0.000103233 |
| **LOC124904895** | + | NC_000020.11:37744397-37747397 | AluSx | + | NC_000020.11:37747096-37747397 | 0 | 2 | No | Yes | -1.67 | 6.76E-11 |
| **LOC130494219** | + | NC_000013.11:109269203-109272203 | LTR9 | + | NC_000013.11:109271795-109272203 | 18 | 48 | Yes | Yes | -1.39 | 2.84E-05 |
| **LOC728715** | - | NC_000012.12:9407414-9410414 | HERVE-int | + | NC_000012.12:9407414-9407886 | 14 | 4 | Yes | Yes | 1.05 | 0.000200187 |
| **LYG1** | - | NC_000002.12:99304775-99307775 | L1MA6 | + | NC_000002.12:99304840-99305292 | 0 | 4 | No | Yes | -2.68 | 0.002761661 |

| | | | | | | | | | | | |
|---|---|---|---|---|---|---|---|---|---|---|---|
| **MYADM** | + | NC_000019.10:53862628-53865628 | AluSx | - | NC_000019.10:53865314-53865624 | 13 | 14 | Yes | Yes | 1.33 | 5.14E-68 |
| **NLRP1** | - | NC_000017.11:5584509-5587509 | AluSx | - | NC_000017.11:5585364-5585636 | 3 | 3 | Yes | Yes | -1.28 | 1.93E-30 |
| **NLRP1** | - | NC_000017.11:5584509-5587509 | MER21C | - | NC_000017.11:5585821-5586370 | 3 | 3 | Yes | Yes | -1.28 | 1.93E-30 |
| **PGLS** | + | NC_000019.10:17508649-17511649 | MIRb | - | NC_000019.10:17508720-17508843 | 0 | 2 | No | Yes | 1.26 | 1.34E-21 |
| **PLGRKT** | - | NC_000009.12:5438377-5441377 | LTR33 | - | NC_000009.12:5439089-5439297 | 4 | 1 | Yes | Yes | 1.50 | 2.65E-31 |
| **PRR7** | + | NC_000005.10:177442995-177445995 | AluSc8 | - | NC_000005.10:177445843-177445965 | 0 | 1 | No | Yes | 1.96 | 6.24E-45 |
| **PSMG3** | - | NC_000007.14:1570032-1573032 | MIR | - | NC_000007.14:1570404-1570543 | 2 | 8 | Yes | Yes | 1.46 | 3.28E-57 |
| **REXO5** | + | NC_000016.10:20803425-20806425 | LTR10C | + | NC_000016.10:20805459-20806036 | 2 | 1 | Yes | Yes | 1.42 | 5.49E-18 |
| **SPMIP6** | - | NC_000009.12:34397810-34400810 | MIR3 | - | NC_000009.12:34397960-34398080 | 0 | 1 | No | Yes | -4.16 | 0.001704811 |
| **UBE2FP1** | - | NC_000003.12:37144182-37147182 | L1ME3Cz | + | NC_000003.12:37144199-37144440 | 8 | 4 | Yes | Yes | -1.54 | 5.86E-13 |
| **UNC119** | - | NC_000017.11:28552628-28555628 | MIRb | - | NC_000017.11:28553407-28553629 | 0 | 3 | No | Yes | -1.02 | 2.13E-26 |
| **ZNF165** | + | NC_000006.12:28077282-28080282 | LTR10A | + | NC_000006.12:28079857-28080282 | 0 | 3 | No | Yes | -2.23 | 2.96E-57 |
| **ZSCAN12P1** | + | NC_000006.12:28087807-28090807 | MIR | - | NC_000006.12:28090713-28090807 | 1 | 9 | Yes | Yes | -2.59 | 3.91E-44 |

**Table S2: Chimeric transcripts detected between genes and transposable elements (TE-terminal type).** Each row lists the gene identifier, strand orientation, and genomic position of the gene, along with the associated TE identifier, strand, and genomic coordinates. Columns report the number of chimeric reads detected in control versus iodine-treated samples, the cell condition where the chimera was observed (Normal or Treated), and differential expression statistics (log2 fold change and adjusted p-value) for the corresponding gene-TE junction.

| gene_id | gene_strand | gene_position | TE_id | TE_strand | TE_position | Chimeric_reads_control | Chimeric_reads_iodine | Normal Cells | Hypoxia | log2FoldChange | padj |
|---|---|---|---|---|---|---|---|---|---|---|---|
| ADORA2B | + | NC_000017.11:15975746-15978746 | AluSx | + | NC_000017.11:15975795-15976095 | 8 | 8 | Yes | Yes | 1.562324723 | 1.61E-56 |
| C2orf74-DT | - | NC_000002.12:61138592-61141592 | Charlie2a | - | NC_000002.12:61141301-61141573 | 0 | 1 | No | Yes | 1.77051957 | 1.94E-19 |
| CARHSP1 | - | NC_000016.10:8849942-8852942 | AluSx | - | NC_000016.10:8850878-8851193 | 2 | 1 | Yes | Yes | 1.764010592 | 3.86E-58 |
| CARHSP1 | - | NC_000016.10:8849942-8852942 | AluSx | - | NC_000016.10:8850878-8851193 | 2 | 1 | Yes | Yes | 1.764010592 | 3.86E-58 |
| COPS7A | + | NC_000012.12:6731865-6734865 | MLT1F2 | - | NC_000012.12:6734457-6734953 | 0 | 1 | No | Yes | 1.518386477 | 3.97E-65 |
| CYP4F11 | - | NC_000019.10:15909377-15912377 | L1MC | + | NC_000019.10:15912140-15912377 | 5 | 20 | Yes | Yes | -2.242939549 | 1.59E-150 |
| DHTKD1 | + | NC_000010.11:12123221-12126221 | L1MA4 | + | NC_000010.11:12125075-12125256 | 1 | 1 | Yes | Yes | 1.456324491 | 1.18E-56 |
| DHTKD1 | + | NC_000010.11:12123221-12126221 | L1MA4 | + | NC_000010.11:12125075-12125256 | 1 | 1 | Yes | Yes | 1.456324491 | 1.18E-56 |
| EPHA2-AS1 | + | NC_000001.11:16166742-16169742 | AluSx3 | - | NC_000001.11:16166781-16166929 | 0 | 1 | No | Yes | 1.703312709 | 7.12E-10 |

| | | | | | | | | | | | |
|---|---|---|---|---|---|---|---|---|---|---|---|
| GATD1 | - | NC_000011.10:764222-767222 | AluJb | + | NC_000011.10:766910-767068 | 7 | 6 | Yes | Yes | 1.563423901 | 1.61E-122 |
| GATD1 | - | NC_000011.10:764222-767222 | AluJb | + | NC_000011.10:766910-767068 | 7 | 6 | Yes | Yes | 1.563423901 | 1.61E-122 |
| GATD1-DT | + | NC_000011.10:784297-787297 | AluSx | + | NC_000011.10:784297-784462 | 0 | 2 | No | Yes | 1.882017385 | 8.71E-07 |
| GINS1 | + | NC_000020.11:25448563-25451563 | L1M5 | - | NC_000020.11:25448563-25448767 | 0 | 2 | No | Yes | 1.562965934 | 2.09E-63 |
| H2BC8 | - | NC_000006.12:26213200-26216200 | AluSx | - | NC_000006.12:26215900-26216148 | 0 | 2 | No | Yes | -3.604406532 | 0.001999 |
| HELB | + | NC_000012.12:66343643-66346643 | L1MB8 | - | NC_000012.12:66343643-66344503 | 0 | 2 | No | Yes | -2.422938798 | 2.37E-59 |
| HERPUD1 | + | NC_000016.10:56944864-56947864 | AluSx1 | + | NC_000016.10:56946166-56946461 | 0 | 1 | No | Yes | -3.445914036 | 1.38E-305 |
| HSP90AA1 | - | NC_000014.9:102077742-102080742 | AluSp | - | NC_000014.9:102080574-102080695 | 0 | 2 | No | Yes | -2.923416836 | 1.53E-279 |
| IFIT1 | + | NC_000010.11:89406487-89409487 | LTR12_v | + | NC_000010.11:89406487-89406836 | 0 | 3 | No | Yes | -3.692741459 | 0.000229 |
| IL32 | + | NC_000016.10:3069530-3072530 | THE1D | - | NC_000016.10:3069593-3069803 | 0 | 1 | No | Yes | -3.212884832 | 7.08E-15 |

| | | | | | | | | | | | |
|---|---|---|---|---|---|---|---|---|---|---|---|
| LINC01186 | - | NC_000023.11:46322924-46325924 | LTR8A | - | NC_000023.11:46324226-46324793 | 23 | 5 | Yes | Yes | 2.056061136 | 1.42E-07 |
| LINC01186 | - | NC_000023.11:46322924-46325924 | THE1B | - | NC_000023.11:46322905-46323280 | 8 | 1 | Yes | Yes | 2.056061136 | 1.42E-07 |
| LINC02328 | + | NC_000014.9:86129778-86132778 | MER61A | + | NC_000014.9:86129778-86129856 | 0 | 2 | No | Yes | -5.539494882 | 9.39E-05 |
| LOC105378626 | - | NC_000001.11:31564938-31567938 | MLT1E3 | + | NC_000001.11:31566396-31567017 | 3 | 2 | Yes | Yes | 1.457915219 | 6.45E-16 |
| LOC105378626 | - | NC_000001.11:31564938-31567938 | MLT1E3 | + | NC_000001.11:31566396-31567017 | 3 | 2 | Yes | Yes | 1.457915219 | 6.45E-16 |
| LOC124901440 | + | NC_000006.12:152839901-152842901 | L1MB1 | + | NC_000006.12:152842673-152843321 | 0 | 3 | No | Yes | -6.450946505 | 9.52E-06 |
| LOC124904428 | + | NC_000001.11:154679835-154682835 | MER5A | - | NC_000001.11:154679910-154680016 | 0 | 1 | No | Yes | -2.07886286 | 2.78E-89 |
| LOC124904895 | + | NC_000020.11:37756386-37759386 | X2_LINE | + | NC_000020.11:37757143-37757314 | 0 | 1 | No | Yes | -2.134347045 | 1.33E-12 |
| METRN | + | NC_000016.10:719655-722655 | AluSx | + | NC_000016.10:719655-719826 | 0 | 1 | No | Yes | 1.957611009 | 5.71E-78 |
| MRPL20-AS1 | + | NC_000001.11:1402046-1405046 | AluSx | - | NC_000001.11:1403846-1404144 | 0 | 1 | No | Yes | 1.614190609 | 1.23E-30 |
| NDRG1 | - | NC_000008.11:133234175-133237175 | MLT1O | + | NC_000008.11:133234328-133234399 | 0 | 1 | No | Yes | -2.614843086 | 1.90E-263 |

| | | | | | | | | | | | |
|---|---|---|---|---|---|---|---|---|---|---|---|
| OXLD1 | - | NC_000017.11:81662036-81665036 | LTR39-int | + | NC_000017.11:81662943-81663400 | 0 | 2 | No | Yes | 1.907784219 | 1.73E-34 |
| PLAUR | - | NC_000019.10:43643095-43646095 | AluJb | - | NC_000019.10:43645944-43646095 | 1 | 17 | Yes | Yes | -2.484144048 | 2.14E-186 |
| PLAUR | - | NC_000019.10:43643095-43646095 | AluJb | - | NC_000019.10:43645944-43646095 | 1 | 17 | Yes | Yes | -2.484144048 | 2.14E-186 |
| S100A2 | - | NC_000001.11:153558108-153561108 | AluSx | + | NC_000001.11:153559863-153560157 | 0 | 3 | No | Yes | 1.49183771 | 8.43E-105 |
| S100A2 | - | NC_000001.11:153558108-153561108 | Charlie18a | + | NC_000001.11:153561004-153561108 | 3 | 4 | Yes | Yes | 1.49183771 | 8.43E-105 |
| S100A2 | - | NC_000001.11:153558108-153561108 | Charlie18a | + | NC_000001.11:153561004-153561108 | 3 | 4 | Yes | Yes | 1.49183771 | 8.43E-105 |
| S100A2 | - | NC_000001.11:153558108-153561108 | MSTD | - | NC_000001.11:153560228-153560580 | 0 | 8 | No | Yes | 1.49183771 | 8.43E-105 |
| SPANXN1 | + | NC_000023.11:145256208-145259208 | L3 | + | NC_000023.11:145256377-145256539 | 0 | 4 | No | Yes | -6.701153713 | 1.84E-06 |
| SPANXN1 | + | NC_000023.11:145256208-145259208 | LTR33 | - | NC_000023.11:145256208-145256376 | 0 | 8 | No | Yes | -6.701153713 | 1.84E-06 |

| | | | | | | | | | | | |
|---|---|---|---|---|---|---|---|---|---|---|---|
| ZFAND 2A | - | NC_000007.14:1145850-1148850 | AluSx | - | NC_000007.14:1148532-1148815 | 1 | 2 | Yes | Yes | -3.122794279 | 6.96e-319 |
| ZFAND 2A | - | NC_000007.14:1145850-1148850 | AluSx | - | NC_000007.14:1148532-1148815 | 1 | 2 | Yes | Yes | -3.122794279 | 6.96e-319 |
| ZFAND 2A | - | NC_000007.14:1145850-1148850 | SVA_E | - | NC_000007.14:1145480-1147951 | 0 | 6 | No | Yes | -3.122794279 | 6.96e-319 |

**Table S3: High-confidence TE–gene chimeric exonization events predicted to trigger nonsense-mediated decay (NMD).** This table lists significantly downregulated genes associated with exonized TE insertions, including TE family identity, log2 fold change, NMD prediction status, and predicted functional impact. All events are classified as high-confidence NMD targets, with SINE (Alu) and LINE insertions predicted to disrupt canonical splicing or introduce premature termination codons.

| gene_id | category | TE_id | log2fc | nmd_prediction | te_impact |
|---|---|---|---|---|---|
| **LOC107986489** | Exonized | AluSc | -5.58 | High confidence NMD target | High - SINE elements often disrupt splicing |
| **CEP192P1** | Exonized | AluJb | -4.53 | High confidence NMD target | High - SINE elements often disrupt splicing |
| **LINC02328** | Exonized | AluSq2 | -5.58 | High confidence NMD target | High - SINE elements often disrupt splicing |
| **OASL** | Exonized | AluYe5 | -5.16 | High confidence NMD target | High - SINE elements often disrupt splicing |
| **OASL** | Exonized | AluJo | -5.16 | High confidence NMD target | High - SINE elements often disrupt splicing |
| **OASL** | Exonized | AluJb | -5.16 | High confidence NMD target | High - SINE elements often disrupt splicing |
| **CEP192P1** | Exonized | L1MC4 | -4.53 | High confidence NMD target | High - LINE elements can introduce premature stops |
| **TMEM217** | Exonized | AluSx1 | -4.99 | High confidence NMD target | High - SINE elements often disrupt splicing |
| **TMEM217** | Exonized | AluSx | -4.99 | High confidence NMD target | High - SINE elements often disrupt splicing |

| | | | | | |
|---|---|---|---|---|---|
| **NCF2** | Exonized | L1ME3A | -4.99 | High confidence NMD target | High - LINE elements can introduce premature stops |
| **LUCAT1** | Exonized | AluY | -4.13 | High confidence NMD target | High - SINE elements often disrupt splicing |
| **LOC100289333** | Exonized | AluSc8 | -4.83 | High confidence NMD target | High - SINE elements often disrupt splicing |
| **LOC105377557** | Exonized | L1MC4a | -4.24 | High confidence NMD target | High - LINE elements can introduce premature stops |
| **LUCAT1** | Exonized | AluSp | -4.13 | High confidence NMD target | High - SINE elements often disrupt splicing |
| **ATG9B** | Exonized | AluSg4 | -4.9 | High confidence NMD target | High - SINE elements often disrupt splicing |
| **RSAD2** | Exonized | AluSg | -5.63 | High confidence NMD target | High - SINE elements often disrupt splicing |
